# Accelerating cryoprotectant diffusion kinetics improves cryopreservation of pancreatic islets

**DOI:** 10.1038/s41598-021-89853-6

**Published:** 2021-05-17

**Authors:** Nikola Dolezalova, Anja Gruszczyk, Kerry Barkan, John A. Gamble, Sam Galvin, Till Moreth, Kevin O’Holleran, Krishnaa T. Mahbubani, Jackie A. Higgins, Fiona M. Gribble, Frank Reimann, Jakub Surmacki, Simon Andrews, John J. Casey, Francesco Pampaloni, Michael P. Murphy, Graham Ladds, Nigel K. H. Slater, Kourosh Saeb-Parsy

**Affiliations:** 1 Department of Surgery, University of Cambridge, Addenbrooke’s Hospital, Hills Road, Box 202, Cambridge, CB2 0QQ UK; 2 Department of Chemical Engineering and Biotechnology, University of Cambridge, Philippa Fawcett Drive, Cambridge, CB3 0AS UK; 3 MRC Mitochondrial Biology Unit, University of Cambridge, Keith Peters Building, Cambridge Biomedical Campus, Hills Rd, Cambridge, CB2 0XY UK; 4 Department of Pharmacology, University of Cambridge, Tennis Ct Rd, Cambridge, CB2 1PD UK; 5 Sosei Heptares, Steinmetz Building, Granta Park, Cambridge, CB21 6DG UK; 6 Institute of Metabolic Science, University of Cambridge, Addenbrooke’s Hospital, Box 289, Cambridge, CB2 0QQ UK; 7 Buchman Institute for Molecular Life Science, Goethe University Frankfurt, Max-von-Laue-Straße 15, 60438 Frankfurt am Main, Germany; 8 Cambridge Advanced Imaging Centre, University of Cambridge, Anatomy Building, Downing Site, Cambridge, CB2 3DY UK; 9 Cavendish Laboratory, University of Cambridge, JJ Thomson Ave, Cambridge, CB3 0HE UK; 10 Bioinformatics Group, The Babraham Institute, Cambridge, CB22 3AT UK; 11 Department of Clinical Surgery, University of Edinburgh, Royal Infirmary of Edinburgh, Edinburgh, EH16 4SA UK; 12 Department of Medicine, University of Cambridge, Addenbrooke’s Hospital, Hills Road, Box 157, Cambridge, CB2 2QQ UK

**Keywords:** Permeation and transport, Diabetes, Diabetes, Translational research

## Abstract

Cryopreservation offers the potential to increase the availability of pancreatic islets for treatment of diabetic patients. However, current protocols, which use dimethyl sulfoxide (DMSO), lead to poor cryosurvival of islets. We demonstrate that equilibration of mouse islets with small molecules in aqueous solutions can be accelerated from > 24 to 6 h by increasing incubation temperature to 37 °C. We utilize this finding to demonstrate that current viability staining protocols are inaccurate and to develop a novel cryopreservation method combining DMSO with trehalose pre-incubation to achieve improved cryosurvival. This protocol resulted in improved ATP/ADP ratios and peptide secretion from β-cells, preserved cAMP response, and a gene expression profile consistent with improved cryoprotection. Our findings have potential to increase the availability of islets for transplantation and to inform the design of cryopreservation protocols for other multicellular aggregates, including organoids and bioengineered tissues.

## Introduction

Despite almost a century of experience with exogenous insulin therapy, diabetes remains a major cause of heart disease, blindness, kidney failure and lower extremity amputation^1–4^. Less than 20% of patients with type 1 diabetes mellitus (T1DM) currently achieve recommended treatment targets necessary to reduce risks of diabetic complications^5^. Whole pancreas transplantation^6^ or pancreatic islet transplantation^7^ offer potential curative treatments but are offered to a very small proportion of (< 0.5%) patients with T1DM^8,9^. Whole pancreas transplantation is a highly invasive surgical procedure with high risk of complications^10^. Transplantation of isolated pancreatic islets^11^ is a less invasive procedure that involves the infusion of only the endocrine islets into the portal vein. Availability of islets is a key limiting factor for access to islet transplantation and, due to an insufficient yield of islets from a single donor, approximately half of patients^11^ require islet infusions from two or more islet donors to achieve insulin independence. Fresh islets can only be cultured for a few days^12^ and lack of a clinically relevant cryopreservation protocol prevents islets isolated from different donors to be preserved and combined. In addition to extending the pool of available islets, effective cryopreservation would enable more time for accurate screening and matching of islets to recipients, and remove some of the logistical challenges of transplantation^13^.

Cryopreservation of cell clusters such as pancreatic islets presents significant additional and unresolved challenges compared to single cells. Pancreatic islets vary largely in size (with average diameter of 109 μm in human)^14,15^ and are composed of densely packed cells. In the absence of perfusion through the vasculature ex vivo, diffusion of solutes into the core of islets necessitates long incubation times. This is problematic if the solute is toxic to cells, as is the case with the commonly used cryoprotectant dimethyl sulfoxide (DMSO)^16^. Furthermore, different cell layers within the islets are subjected to unequal cryoprotectant and intracellular water concentrations, as well as to non-uniform temperature changes during cryopreservation, which can lead to increased and differential ice crystal formation^17^.

One strategy to achieve homogeneous exposure of islet cells to DMSO during cryopreservation is the sequential incubation of islets with different DMSO concentrations for different durations (e.g., in 0.67 M DMSO for 5 min at 22 °C, followed by incubation in 1 M DMSO for 25 min at 22 °C and final incubation in 2 M DMSO for 15 min at 0 °C before freezing)^18^. Studies on diffusion of DMSO into islets suggest that these incubation times may be sufficient for equilibration into the core of islets^19,20^. However, there is limited data on diffusion rates of other cryoprotectants, including non-penetrating cryoprotectants such as sugars. Another strategy to improve cryopreservation is the use of a combination of cryoprotectants to reduce exposure to toxic concentrations^21^. Trehalose and other non-penetrating cryoprotectants have been used as additives for cryopreservation of a variety of single cells^22–24^ and islets^25,26^, although it is not clear whether these protocols resulted in enhanced cryoprotection compared to DMSO alone. Despite their potential, these strategies have not led to the development of a clinically used islet cryopreservation protocol, which remains an unmet need. Importantly, a clinically-relevant protocol for islet cryopreservation would have potential applications for preservation of other multicellular aggregates such as organoids and bioengineered tissues^27^.

In this study, we developed a method to determine diffusion kinetics of small molecules in aqueous solutions into the core of pancreatic islets and to investigate strategies to accelerate it. Based on these results, we designed a cryopreservation protocol which combines DMSO with trehalose pre-incubation to achieve improved post-thaw viability.

## Results

### Standard incubation times are not sufficient to achieve solute equilibration

**Figure 1:**
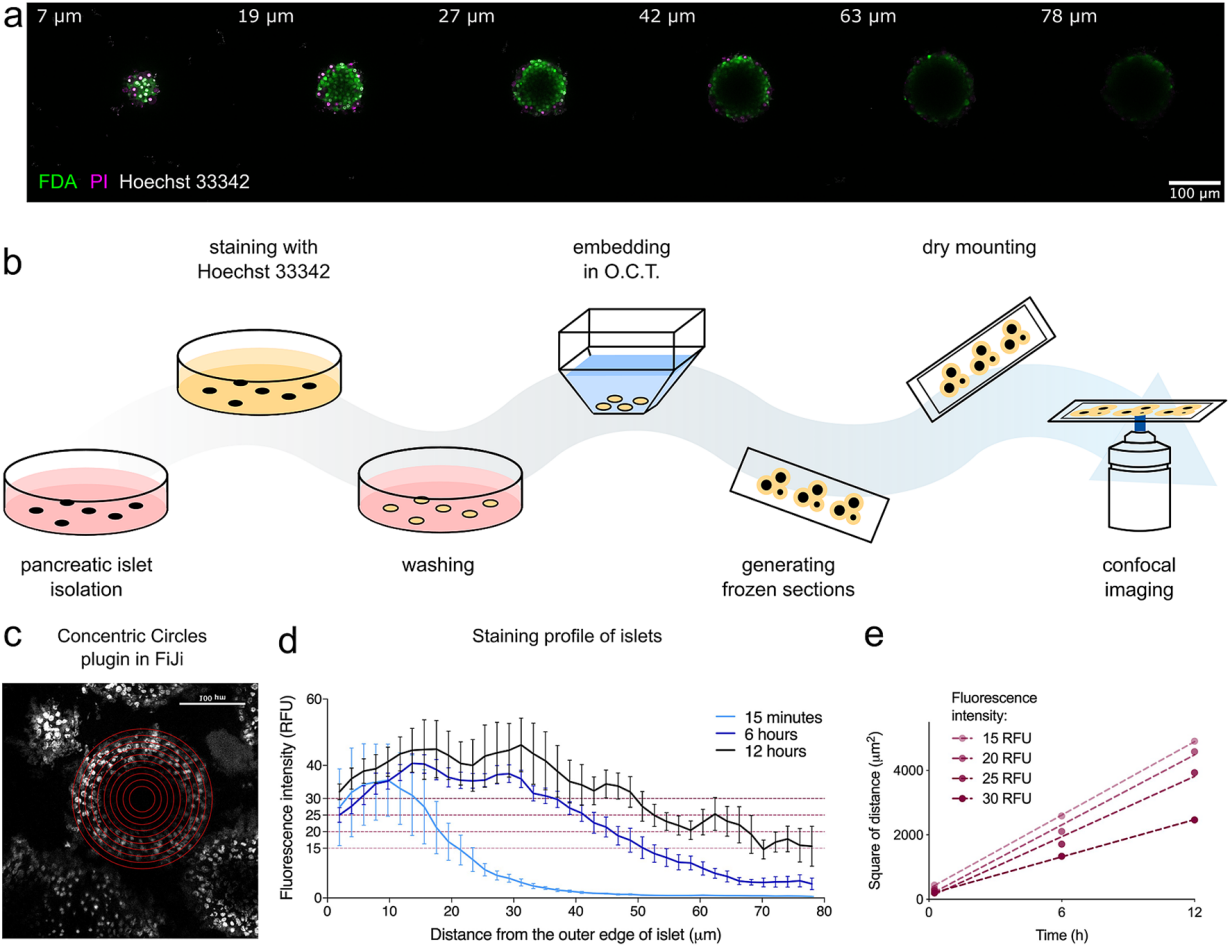
Molecular diffusion into islets—imaging and method development. (**a**) Lateral optical cross-sections of a pancreatic islet on top of a coverslip (unflattened) imaged by a confocal microscope (FDA/PI/Hoechst 33342 staining for 15 min at room temperature); each slice is labelled with distance in μm from the top of the islet. (**b**) Novel method to study diffusion: isolated pancreatic islets were incubated with Hoechst 33342 nuclear stain under different conditions, washed and immediately embedded into Optimal Cutting Temperature compound (O.C.T.) to stop the diffusion of the dye further into the islet. Frozen sections were cut on a cryostat and coverslip was added using a dry mounting method (double-sided tape) to prevent any exposure to moisture. Each slide was imaged immediately after being thawed to prevent further diffusion of the dye into the islet. (**c**) Confocal images of the sectioned islets were analysed using ImageJ plugin Concentric Circles. Parameters of the plugin were set to align with the outer edge of the imaged islet and to draw circles 5 pixels (corresponding to 1.89 μm) apart. Average intensity of each circle was measured, and results were plotted to show a staining profile. (**d**) Average staining profiles of pancreatic islets incubated with Hoechst 33342 at room temperature for 15 min, 6 h or 12 h (n = 3–5 islets). To get an average staining profile for a certain staining condition, profiles from multiple islets were aligned at the outer edge (0 μm). (**e**) Linear relationship between time and square of distance shown for four different fluorescent intensities from plot shown in (**c**). Figure (**b**) was created using Inkscape (version 0.91, http://www.inkscape.org/), figures (**d**) and (**e**) using GraphPad Prism (version 8.4.1, https://www.graphpad.com). *RFU* relative fluorescence intensity.

We first quantified diffusion kinetics of solutes in aqueous solutions into the core of mouse pancreatic islets. Clinical assessment of islet viability typically involves dual fluorescent staining with fluorescein diacetate (FDA) and propidium iodide (PI) for 15 min or less at room temperature (Supplementary Fig. 1). Using this protocol, we observed good staining of peripheral cells on laser scanning confocal microscopy, but little or no signal from cells located deeper than 20–30 μm from the periphery of islets (Fig. 1a). As both diffusion kinetics and imaging limitations could have contributed to lack of staining of the islet core, we developed a method to investigate the need for longer incubation of islets with small molecules. We incubated islets with Hoechst 33342 dye, which is resistant to photo-bleaching and stains all cells irrespective of their viability, for different durations, followed by arrest of diffusion by freezing (Fig. 1b). Frozen sections of the dye-loaded islets thus enabled measurement of the depth of diffusion of the dye from the surface of the islets (Fig. 1c). The intensity of staining towards the central core of islets increased with prolonged incubation, but the core of the islets remained minimally stained even after 12 h of incubation at room temperature (Fig. 1d). The intensity of staining was in line with Fick’s law (Fig. 1e), which states that the square of the distance of the penetration by diffusion increases linearly with the time allowed for penetration^28^. This correlation is consistent with the hypothesis that molecular diffusion is primarily responsible for the movement of dye molecules in this system.

### Increasing temperature can accelerate solute diffusion

**Figure 2:**
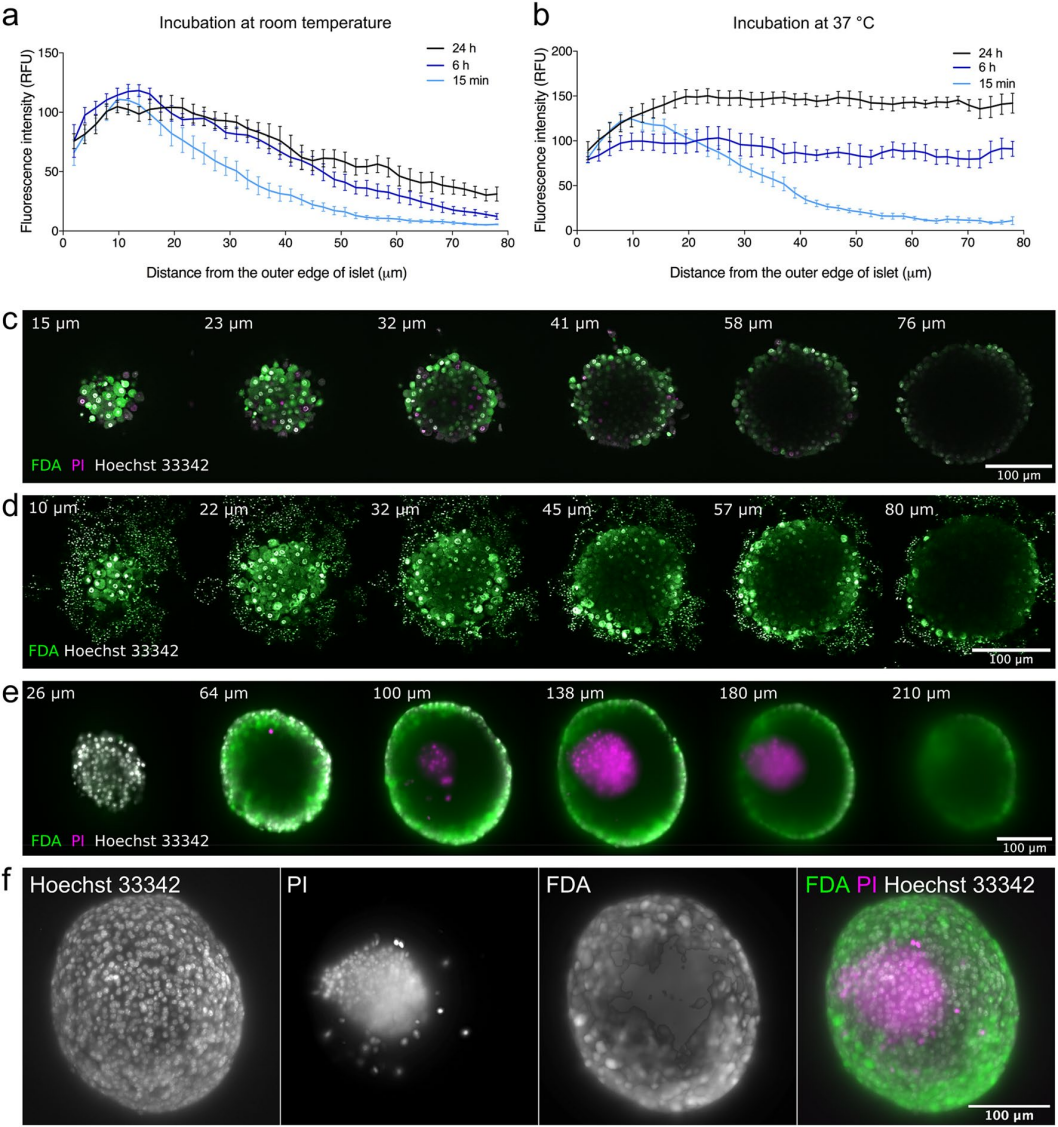
Effect of temperature on diffusion of nuclear dye Hoechst 33342 into pancreatic islets. (**a**) Staining profiles of pancreatic islets incubated with Hoechst 33342 for 15 min, 6 h or 24 h at room temperature (n = 6–7). (**b**) Staining profiles of pancreatic islets incubated with Hoechst 33342 for 15 min, 6 h or 24 h at 37 °C (n = 6–7). (**c**) Pancreatic islet stained with FDA (green), PI (magenta) and Hoechst 33342 (white) for 6 h at 37 °C imaged a confocal microscope. (**d**) Pancreatic islet stained with FDA (green) and Hoechst 33342 (white) for 6 h at 37 °C imaged on a two-photon microscope. (**e**) Pancreatic islet stained with FDA (green), PI (magenta) and Hoechst 33342 (white) for 6 h at 37 °C imaged on a light sheet microscope. (**f**) 3D-reconstruction from multi-angle imaging (4 angles, 90° apart) of pancreatic islet stained with FDA (green), PI (magenta) and Hoechst 33342 (white) using light sheet microscopy. Representative images shown. Figures (**a**) and (**b**) were produced in GraphPad Prism (version 8.4.1, https://www.graphpad.com). In (**c**–**a**), each slice is labelled with distance in μm from the top of the islet. *FDA* fluorescein diacetate, *PI* propidium iodide.

Incubation times > 12 h present a practical obstacle to assessment of viability and cryopreservation of islets in clinical setting. We therefore investigated strategies to accelerate diffusion into the core of pancreatic islets. Increasing hydrostatic pressure through centrifugation resulted in an elevated concentration of dye molecules on the surface of the islets but only a marginal increase in the depth of diffusion (Supplementary Fig. 2a,b). DMSO is known to have high diffusivity^19^ and its properties as an enhancer of penetration across membranes is well described^29^. Although 10% (but not 1%) DMSO enhanced the diffusion rate significantly (Supplementary Fig. 2c–f), it led to significant necrosis-related karyolysis at the periphery of the islets (Supplementary Fig. 2g,h). We finally investigated the effect of temperature on diffusion of Hoechst 33342 in islets. At room temperature, staining of the islet core was much lower than the periphery even after 24 h of incubation (Fig. 2a). At 37 °C, however, islets were homogeneously stained after only 6 h of incubation (Fig. 2b, Supplementary Fig. 2i). Importantly, viability of the islets incubated with the dye for 6 h at 37 °C remained above 90% (Supplementary Fig. 2j). We next imaged pancreatic islets after incubation with viability dyes for 6 h at 37 °C. The optical properties of the islets prohibited adequate imaging of the core of the islets using scanning confocal microscopy (Fig. 2c) or two-photon excitation microscopy (Fig. 2d). However, light-sheet microscopy (Fig. 2e,f) clearly demonstrated the presence of dead cells at the core of islets while the periphery consisted of viable cells.

### Pre-incubation with trehalose improves post-thaw islet survival

**Figure 3:**
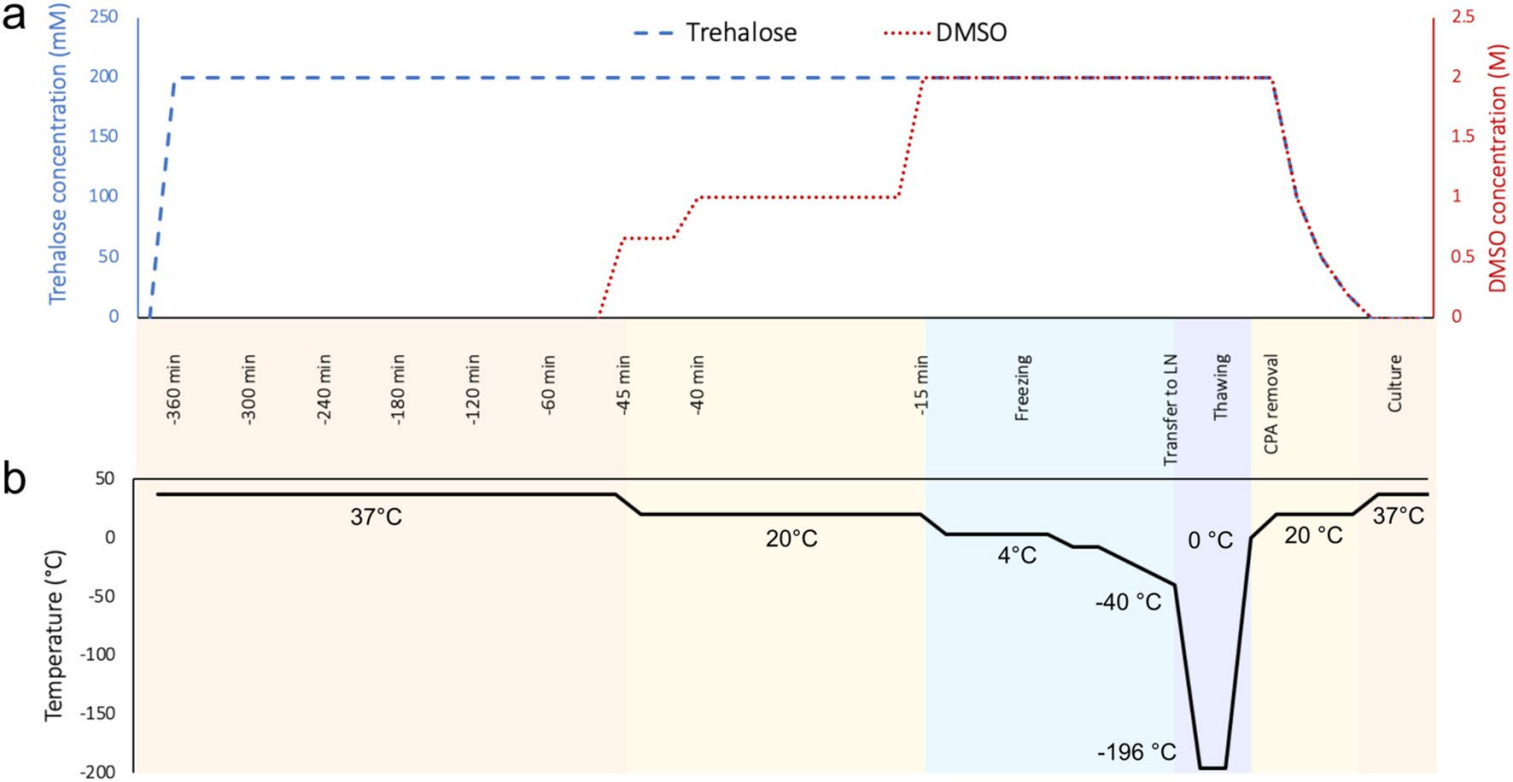
Schematic of the proposed islet cryopreservation protocol. (**a**) Changes in concentrations of cryoprotectants in the cryopreservation solution over time. (**b**) Changes in temperatures of the cryopreserved sample over time of the cryopreservation (time of freezing is t = 0 min). Islets are preincubated in 200 mM trehalose at 37 °C for 6 h (trehalose is kept at stable concentration throughout the incubation). 45 min before the end of this incubation time, step-wise DMSO addition protocol is initiated and samples are frozen in a programmable freezer. Frozen samples are plunged into liquid nitrogen and stored in a liquid nitrogen dewar until thawing. Thawing is performed in a 37 °C water bath until sample reaches 0 °C and CPAs are removed using a step-wise protocol. Thawed islets are transferred into a fresh culture medium and can be cultured at 37 °C. *CPA* cryoprotectant, *LN* liquid nitrogen.

**Figure 4:**
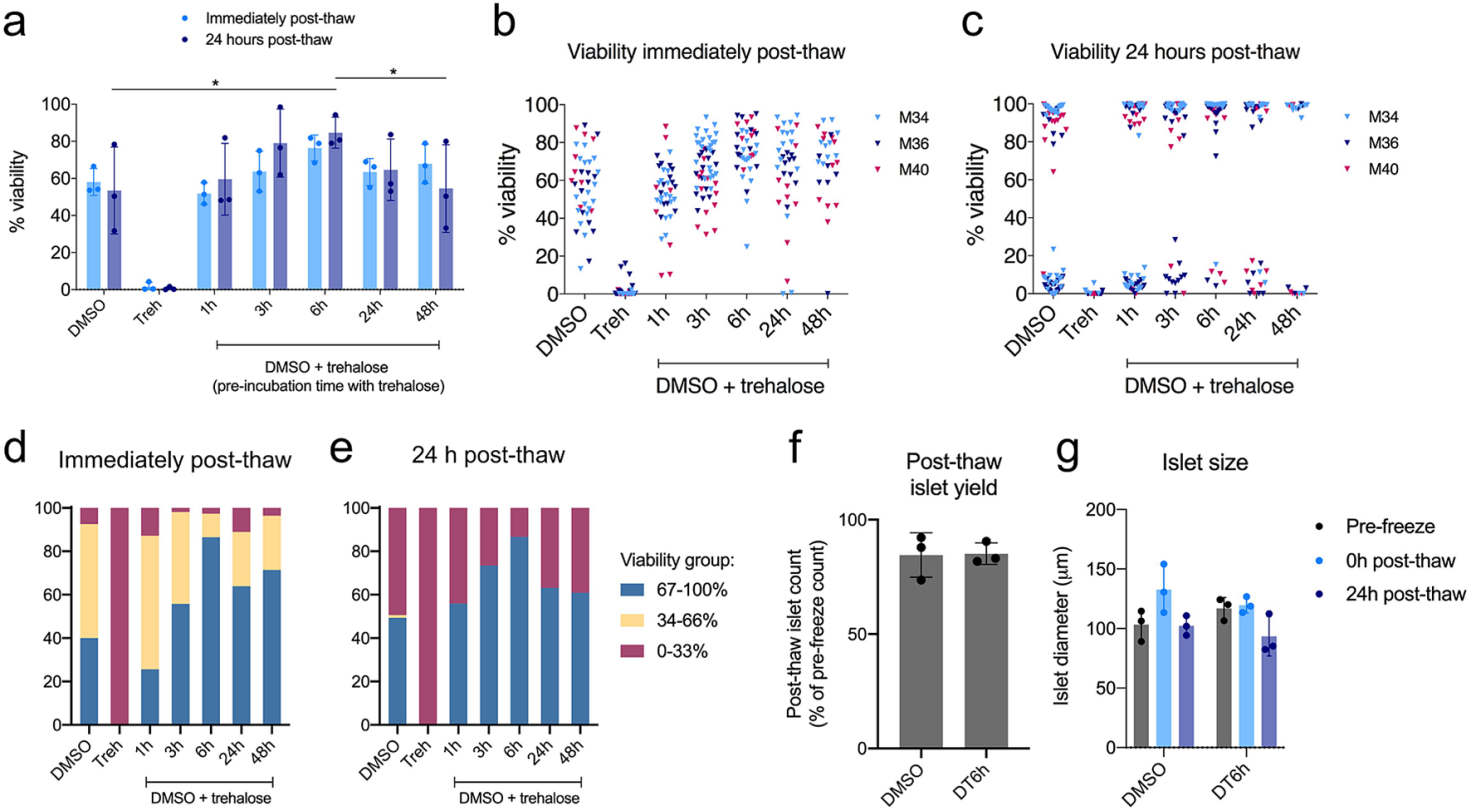
Viability, size and number recovered using the proposed islet cryopreservation protocol. (**a**) Post-thaw viability of pancreatic islets cryopreserved with proposed DMSO + trehalose protocol with varying trehalose pre-incubation time (1–48 h). Viability measured immediately post-thaw (light blue bars) and 24 h post-thaw (dark blue bars) shown. Bars represent mean ± SD, n = 3 islet isolations, repeated measures two-way repeated measures ANOVA followed by Tukey’s HSD post-hoc test, all groups were significantly different from trehalose only (Treh), other significant differences (p ≤ 0.05) are marked by asterisks. (**b**) Post-thaw viability of individual islets from the experiments shown in (**a**) measured immediately post-thaw and (**c**) 24 h post-thaw. Triangles represent individual islet viabilities; triangles of same colour represent results from the same experiment. (**d**) Distribution of islets from (**a**) in three viability groups for DMSO- and DT6h-cryopreserved islets immediately post-thaw and (**e**) 24 h post-thaw. (**f**) Post-thaw islet yield for islets cryopreserved with DMSO or DT6h, calculated as number of islets recovered after thawing/number of islets before freezing. (**g**) Average islet diameter of DMSO- and DT6h-cryopreserved islets measured pre-freeze, 0 h post-thaw and 24 h post-thaw. All figures were produced using GraphPad Prism (version 8.4.1, https://www.graphpad.com). *DT6h* DMSO + trehalose pre-incubated for 6 h, *Treh* trehalose only.

To develop a new cryopreservation method for islets, we first optimised published freezing^30^ and thawing^31^ protocols using DMSO alone (Supplementary Fig. 3a–c). As expected, freezing with the non-toxic cryoprotectant trehalose alone did not provide sufficient cryoprotection, regardless of incubation time and trehalose concentration (Supplementary Fig. 3d,e). We therefore next explored combining pre-incubation with trehalose followed by addition of DMSO as a cryopreservation method. After evaluating the toxicity of trehalose at a range of concentrations (Supplementary Fig. 3f), we used the highest non-toxic concentration of trehalose (200 mM). To develop the protocol, we pre-incubated islets with trehalose for 1, 3, 6, 24 or 48 h at 37 °C, added DMSO in the last 45 min and cryopreserved islets using a controlled-rate freezer (Fig. 3). Pre-incubation with trehalose for 6 h (DT6h group) resulted in significant improvement of viability 24 h after thawing compared to DMSO alone (Fig. 4a). Individual islet viabilities displayed the expected distribution immediately after thawing (Fig. 4b) but shifted towards a bimodal distribution at 24 h, with islets separating into two groups: viability of some islets was close to 100% and the remainder close to the minimum of 0% (Fig. 4c). There was a significant positive correlation between the islet size and viability at 24 h post-thaw in the DT6h group and at both time points in the DMSO group (Supplementary Fig. 4a,b). The DT6h group yielded the most islets in the 67–100% viability group at both times of measurement (Fig. 4d,e). There was no difference in post-thaw islet yield (number of islets recovered) and islet size in the DT6h group compared to cryopreservation with DMSO only (Fig. 4f,g, Supplementary Fig. 4c for raw data). Post-thaw islet morphology was maintained in both groups, with DT6h showing less debris detected on the surface of islets or in the free medium at 24 h after thawing (Supplementary Fig. 4d).

### The novel protocol maintains ATP/ADP ratios and cyclic AMP response

**Figure 5:**
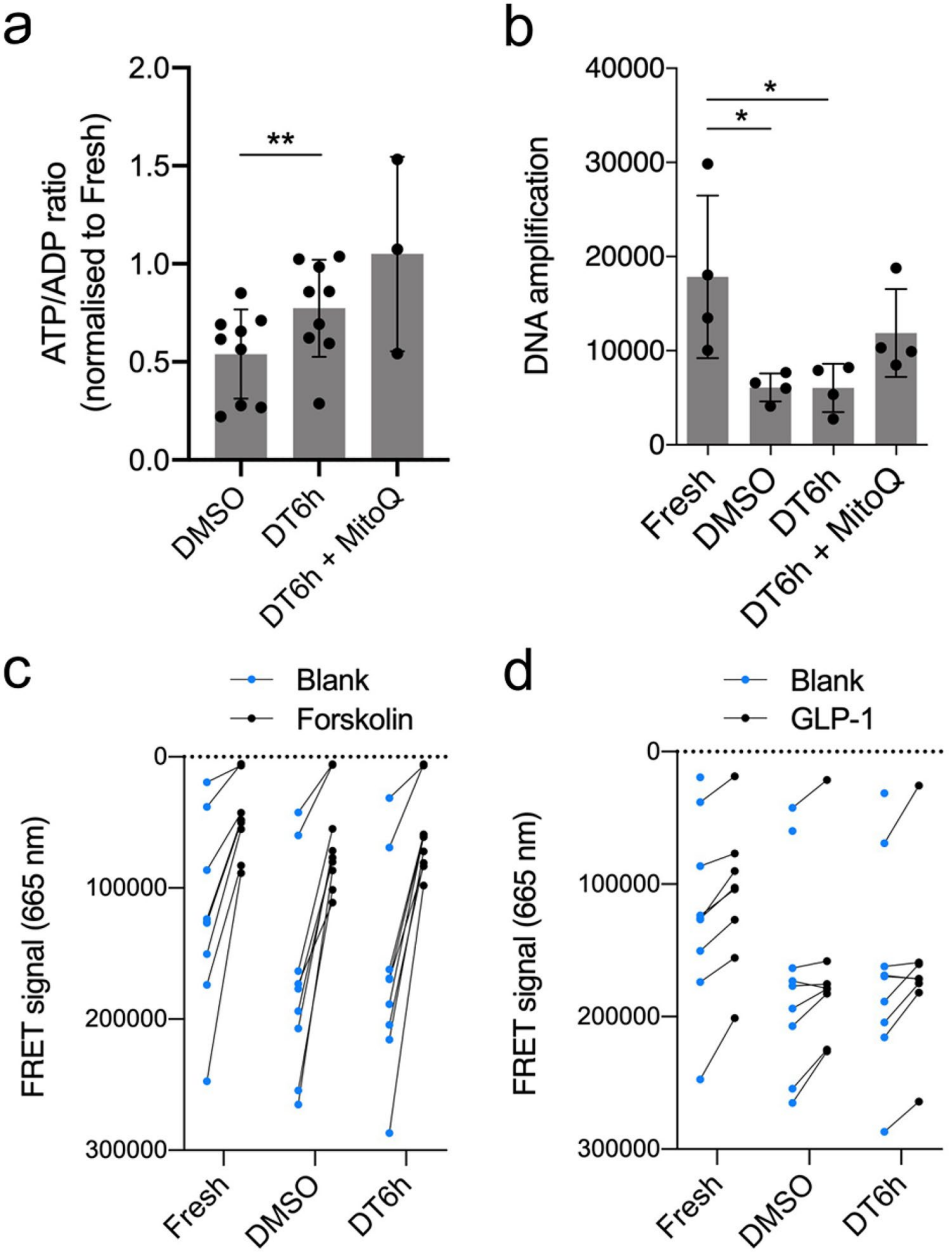
Metabolic data for islets cryopreserved using the proposed protocol (DT6h). (**a**) ATP/ADP ratio of cryopreserved islets 24 h post-thaw normalised to ATP/ADP ratio in fresh islets (n = 9 islet isolations). (**b**) Mitochondrial DNA oxidation levels in fresh, DMSO-cryopreserved and DT6h-cryopreserved islets. Higher amplification numbers indicate lower oxidation of mitochondrial DNA (n = 4 islet isolations). (**c**) Cyclic AMP (cAMP) production in fresh, DMSO-cryopreserved and DT6h-cryopreserved islets upon exposure to forskolin or (**d**) GLP-1. Data in (**c**) and (**d**) shown as raw values of the fluorescence resonance energy transfer (FRET) signal at 665 nm, which is inversely proportional to the cAMP concentration in the samples. Data obtained from the same islet sample are connected with a line (n = 8–9 islet isolations). Missing data were below the detection level of the assay. Repeated measures one-way ANOVA followed with Tukey’s HSD post-hoc test was performed, statistically significant differences are marked by asterisks: *p ≤ 0.05, **p ≤ 0.01. All figures were produced using GraphPad Prism (version 8.4.1, https://www.graphpad.com).

Mitochondrial markers are recognise as important parameters in evaluation of pancreatic islet health and are currently one of the most reliable predictors of in vivo islet function^32,33^. Relative to control fresh islets, cryopreservation using the DT6h protocol maintained ATP/ADP ratio better than DMSO (77.3 ± 24.7% vs. 54.0 ± 22.7%; p = 0.0057, Fig. 5a). In both cryopreserved conditions, there was a significant decrease in the absolute concentration of adenosine nucleotides, likely caused by the loss of viable cells during cryopreservation (Supplementary Fig. 4e). Consistent with a key role for reactive oxygen species and mitochondrial injury during cryopreservation, the addition of the mitochondria-targeted antioxidant MitoQ^34^ to the DT6h protocol further improved ATP/ADP ratio to a level comparable to fresh islets (Fig. 5a). Both DMSO- and DT6h-cryopreserved islets showed increased DNA damage (manifested as reduced amplification^35,36^), which could be partially rescued by the addition of MitoQ (Fig. 5b). The ATP/ADP ratios and mitochondrial DNA oxidation levels were reflected in the post-thaw viability scores in the three groups (Supplementary Fig. 4f). To examine the integrity of intracellular pathways regulating insulin secretion, we next investigated activation of adenylyl cyclase and production of cyclic AMP (cAMP)^37^ in fresh and cryopreserved islets. cAMP production induced by the adenylate cyclase activator forskolin (Fig. 5c) and incretin hormone GLP-1 (Fig. 5d) was comparable in fresh, DMSO- and DT6h-cryopreserved islets, suggesting that this pathway remains intact after cryopreservation using both methods.

### The novel protocol maintains peptide secretion from β-cells

**Figure 6:**
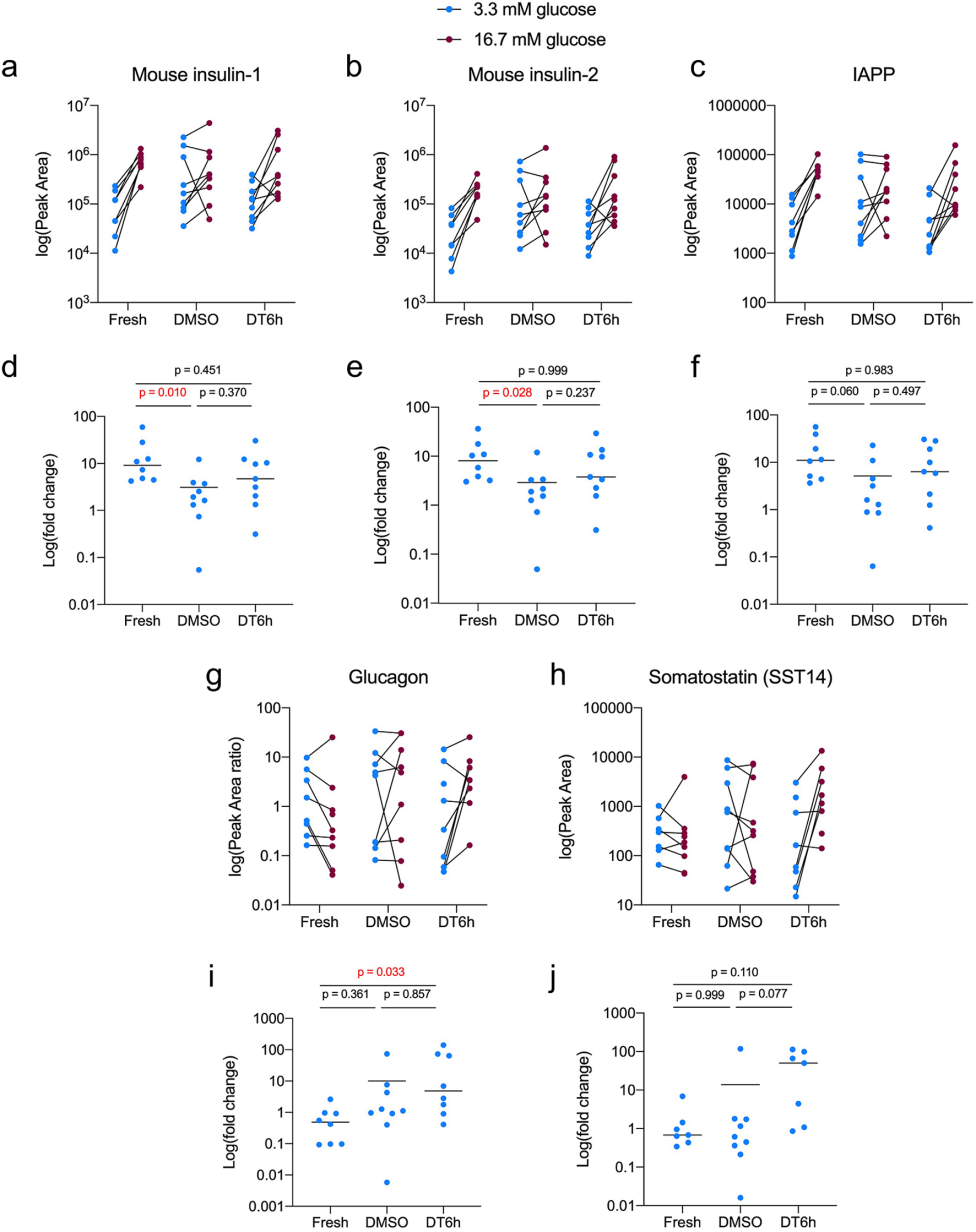
Peptide production by islets after glucose stimulation. Peptide levels measured by LC–MS/MS for (**a**) mouse insulin-1, (**b**) mouse insulin-2 and (**c**) islet amyloid polypeptide (IAPP). Fold changes calculated as the ratio of Peak Area measured in 16.7 mM glucose and in 3.3 mM glucose for (**d**) mouse insulin-1, (**e**) mouse insulin-2, (**f**) islet amyloid polypeptide (IAPP). Peptide levels measured by LC–MS/MS for (**g**) glucagon and (**h**) somatostatin (SST-14). Fold changes calculated as the ratio of Peak Area concentration measured in 16.7 mM glucose and in 3.3 mM glucose for (**i**) glucagon and (**j**) somatostatin (SST-14). Peak Areas for glucagon were normalised to Peak Areas for glucagon internal standard. One-way Kruskal–Wallis analysis of variance was performed for the fold changes followed by Dunn’s correction for multiple comparisons (n = 9 samples from 3 isolations, each sample = 10 islets; p-values are shown for each comparison, significant differences are highlighted in red). All figures were produced in GraphPad Prism (version 8.4.1, https://www.graphpad.com).

Using mass spectrometry-based peptidomics, we next screened peptide secretion by pancreatic islets exposed to two glucose concentrations. Both DMSO- and DT6h-cryopreserved islets remained responsive to increased glucose concentration by increasing the release of insulin (Fig. 6a,b) and IAPP (islet amyloid polypeptide) which is co-stored and released with insulin (Fig. 6c). DMSO-cryopreserved islets showed a significantly lower fold-change in insulin release for both insulin-1 and insulin-2 (duplicate genes in mouse genome, Fig. 6d,e) and dysregulated release of IAPP (Fig. 6f). DT6h showed an insulin and IAPP release profile more similar to fresh islets, with low insulin release in 3.3 mM glucose and its upregulation in 16.7 mM glucose (Fig. 6a,b). Glucagon and somatostatin were detected in very low quantities in media from all three groups**.** Both DMSO- and DT6h-cryopreserved islets showed highly variable secretion profiles (Fig. 6g,h) and fold changes for both peptides (Fig. 6i,j).

### Transcriptomic differences in insulin production and stress response pathways

**Figure 7:**
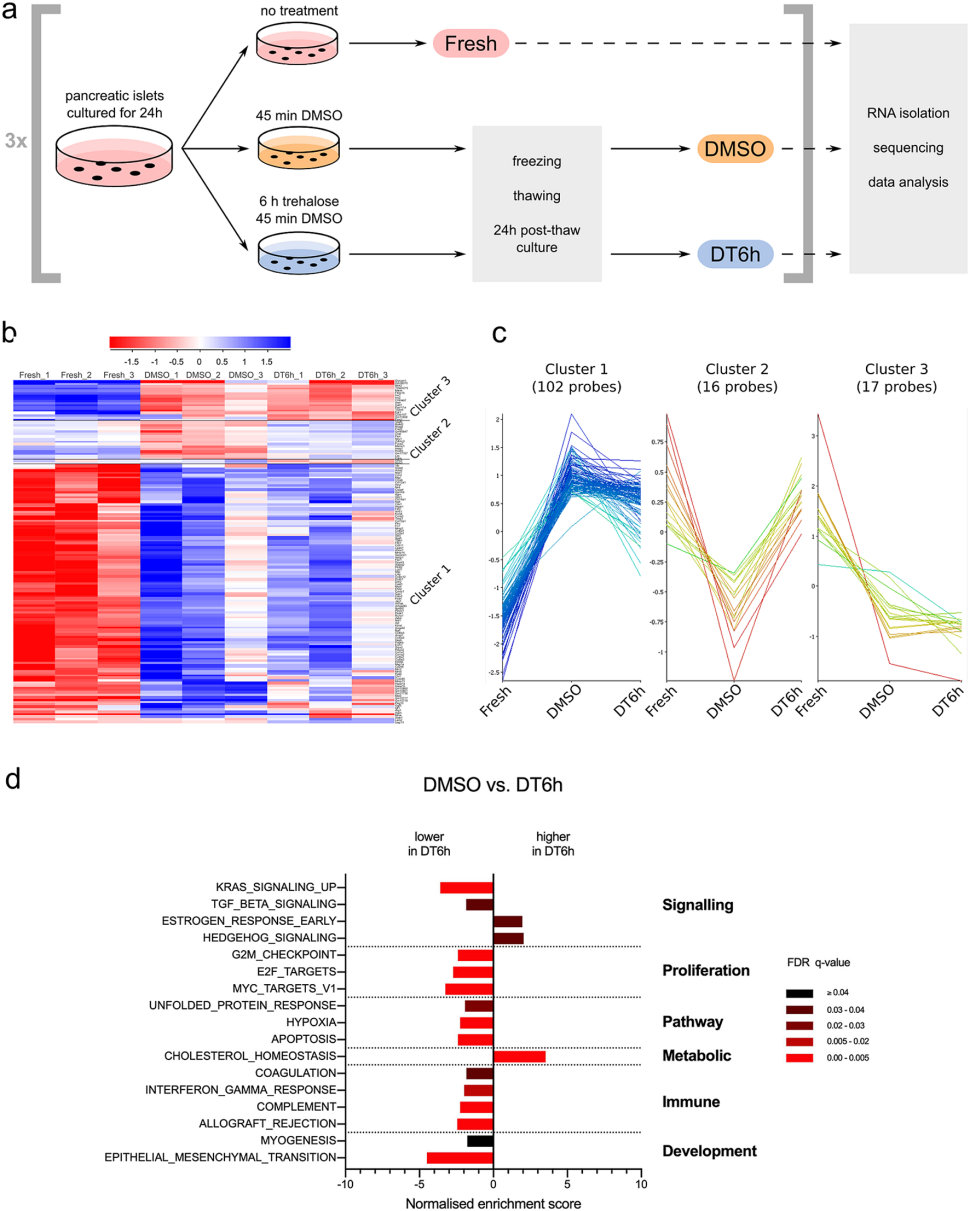
Impact of cryopreservation on islet transcriptome. (**a**) Schematic representation of the experimental design. Islets from three separate isolations (n = 3) were used as shown and samples were stored in RNAlater solution. RNA from all samples was processed and sequenced on the same occasion. (**b**) Heatmap showing the 142 genes that are differentially expressed between the three tested conditions (genes common to both the DESeq2 and Intensity Difference analysis, FDR ≤ 0.05, n = 3 islet isolations, individual replicates shown), clustered based on the hierarchical cluster analysis. (**c**) Line graph representation of the gene clusters 1, 2 and 3, re-plotted from (**b**). (**d**) Preranked Gene Set Enrichment Analysis (GSEA-Preranked) of relative differences in Hallmark gene set expression levels between DMSO- and DT6h-cryopreserved islets (differences of FDR ≤ 0.05 are shown). The gene sets are grouped according to the Process Categories of the Molecular Signatures Database Hallmark Gene Sets (see “Methods”). FDR q-values are highlighted in different shades of red. Figure (**a**) was created using Inkscape (version 0.91, http://www.inkscape.org/), figures (**b**) and (**c**) using SeqMonk^39^ (version 1.45.4) and figure (**d**) using GraphPad Prism (version 8.4.1, https://www.graphpad.com).

We compared RNA isolated from fresh, DMSO- or DT6h-cryopreserved islets as outlined in Fig. 7a. DESeq2^38^ comparison to fresh islets revealed 2423 and 2870 differentially expressed genes in the DMSO and DT6h groups, respectively. Heatmap visualisation of a three-group comparison using DESeq2 shows the differences between fresh and frozen samples but also a level of variability between the frozen sample replicates (Supplementary Fig. 5). A second analysis of differential gene expression, using the Intensity Difference filter native to SeqMonk^39,40^, was then carried out to identify genes with the highest fold-change relative to their expression level. There was an overlap of 142 differentially expressed genes between these two analyses, clustering into three main gene clusters (Fig. 7b,c). Of these, Clusters 1 and 3 show genes which were up- or down-regulated in both cryopreserved groups when compared to fresh, and Cluster 2 consists of 16 genes which were down-regulated in DMSO but showed similar level in fresh and DT6h groups. These included genes that are involved in the uptake and metabolism of cholesterol (*Insig1*, *Fdps*, *Pcsk9*, *Lss*, *Cln6*, *Msmo1* and *Vegfb*), stress responders and regulators of apoptosis (*Vegfb*, *Pcsk9*, *Flcn* and *Fnip2*).

Preranked Gene Set Enrichment Analysis (GSEA-Preranked) showed large differences between fresh and cryopreserved islets, with ten gene sets downregulated and 27 upregulated in DMSO and nine downregulated and 24 upregulated in DT6h (Suppl. Fig. 6a,b). Both cryopreserved groups showed downregulation of the ‘protein secretion’ and ‘pancreatic beta cell’ signatures which is in line with *Ins1* and *Ins2* being identified as the most down-regulated genes in both cryopreserved conditions when compared to fresh (− 6.80 and − 6.83 fold change for DMSO, − 6.59 and − 5.96 fold change for DT6h). GSEA comparison of DMSO and DT6h showed fewer differences, with 14 gene sets showing lower and three gene sets higher relative expression in DT6h (Fig. 7d). Relative to the DMSO group, the DT6h group showed decreased expression of proliferation, pro-inflammatory, hypoxia- or apoptosis-related gene sets, among others. Conversely, the ‘cholesterol homeostasis’ gene set showed higher expression in the DT6h dataset than in DMSO. The above gene sets followed the same direction of change for both DMSO and DT6h when compared to the fresh group. Similar results were produced by pathway analysis against the more granular Gene Ontology: Biological Process gene set. There was an upregulation in vesicular transport, lipid metabolism and response to insulin in the DT6h group, and a reduction in oxidative stress, immune activation and cell differentiation (Supplementary Fig. 7). Of note, there were no differences in insulin exocytosis pathways (Supplementary Table 2).

### In vivo performance of cryopreserved islets remains variable

**Figure 8:**
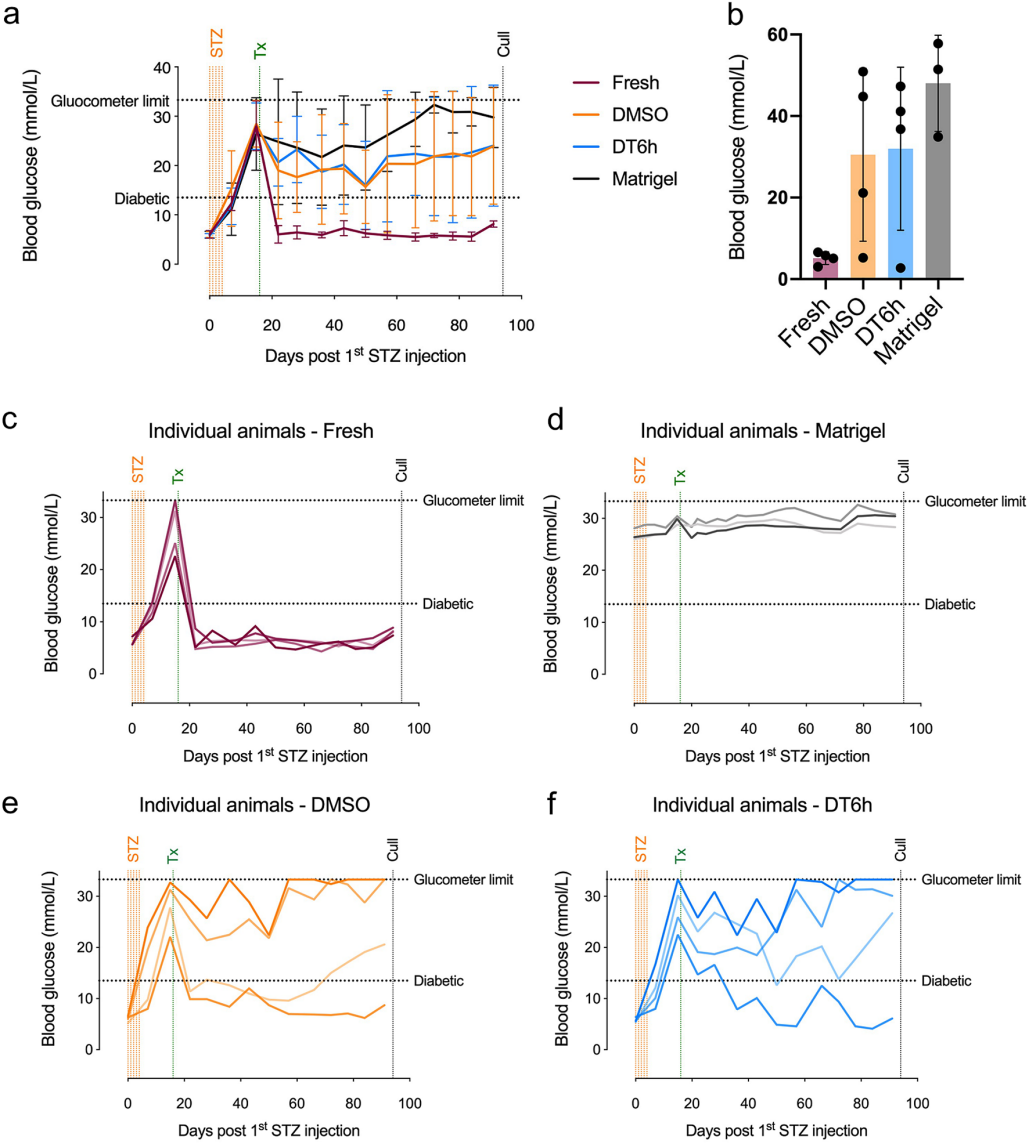
Engraftment of cryopreserved islets in STZ-induced diabetes mouse model. (**a**) Fasting blood glucose measurements in diabetic mice transplanted with 800 fresh, DMSO-cryopreserved or DT6h-cryopreserved islets or vehicle (Matrigel), measured using portable glucose meter (n = 3–4 mice). (**b**) Fasting blood serum glucose measured using an immunoassay on day 75 days after transplantation (day 91 after first STZ injection). (**c**–**f**) Fasting blood glucose in individual diabetic mice transplanted with (**c**) 800 fresh islets, (**d**) Matrigel/vehicle only, (**e**) 800 DMSO-cryopreserved islets and (**f**) 800 DT6h-cryopreserved islets over the time course of the experiments. All plots were produced using GraphPad Prism (version 8.4.1, https://www.graphpad.com). *STZ* streptozotocin, *Tx* transplantation.

To evaluate the performance of the two cryopreservation techniques in vivo, we transplanted 800 fresh, DMSO- and DT6h-cryopreserved islets under the kidney capsule of streptozotocin-induced diabetic mice. The dose of 800 islets was chosen over lower doses (300 or 400 islets) because it reliably restored normoglycaemia in all mice transplanted with fresh islets (Supplementary Fig. 8). There was no difference in the blood glucose levels between the DMSO and DT6h groups. Both groups showed higher glucose levels over time than mice transplanted with fresh islets but lower levels than animals transplanted with the vehicle (Matrigel) only (Fig. 8a). At the end of the experiment, the average blood glucose levels were 5.1 ± 1.5 for fresh islets, 30.5 ± 21.2 for DMSO-cryopreserved islets, 32.0 ± 20.0 for DT6h-cryopreserved islets and 48.0 ± 11.8 for Matrigel (Fig. 8b). In the group transplanted with fresh islets, all mice achieved normoglycaemia by 6 days post-transplantation. The two cryopreserved groups showed high variability between animals (Fig. 8c–f).

### Human pancreatic islet cryopreservation protocol needs further optimisation

We performed a pilot study to evaluate whether the cryopreservation protocol developed for mouse islets could be directly translated to human. Islets from three human donors (Supplementary Table 1) showed a post-thaw viability of ~ 60% immediately and ~ 40% at 24 h post-thaw in all cryopreserved conditions except with no cryoprotectant which was close to 0%. However, a clear benefit of trehalose addition and the trend between the different trehalose pre-incubation times (1, 3 or 6 h) shown for mouse islets (Fig. 4a) was not be observed for human islets in these preliminary experiments (Supplementary Fig. 9).

## Discussion

In this study, we describe a method for measuring diffusion rates for small molecules in aqueous solutions into the core of non-vascularised tissue using Hoechst 33342 dye. While this serves as a useful approximation, other small molecules, including trehalose and viability dyes, may follow different diffusion rates based on their concentration gradient or ability to cross the membrane. Other properties modulating the diffusion rate of molecules include molecular weight, charge or hydrophobicity and therefore, empirical evidence should be obtained for each cryoprotectant of interest to adjust any estimates or approximations. Diffusion of Hoechst 33342 into the islet core was accelerated from > 24 to 6 h by increasing temperature to 37 °C, without detrimental effect on viability. In addition to utility in islet cryopreservation, this approach can be used for clinically relevant islet transplant pre-conditioning protocols, such as incubation with antioxidants to ameliorate oxidative damage. RNA sequencing revealed upregulation of hypoxia-related genes in frozen islets, suggesting significant oxidative stress during cryopreservation. As proof-of-concept, we utilised mitochondrial-targeted antioxidant MitoQ to reduce mitochondrial injury and demonstrated improved post-thaw viability and mitochondrial health. While these experiments support a role for oxidative damage during cryopreservation, further studies will be required to systematically test the efficacy of MitoQ to enhance cryopreservation of pancreatic islets.

Our findings suggest that current clinical viability staining protocols fail to assess the viability of the islet core. Because central necrosis of cultured islets is common^41^, viability of islet batches is often overestimated and does not correlate with function^42^. In this study, we identified two limitations of current viability assessment: insufficient staining times and inability to image the core of islets due to challenging optical properties of islets^43^. Although light sheet fluorescence microscopy was the most successful technique in imaging the islet core, its use in clinical assessment is hampered by practical and logistic challenges. Strategies to overcome these challenges include flattening islets using the method described in Supplementary Fig. 1 (for high-throughput screening) or the use of dyes emitting at longer wavelengths (red/near infrared dyes) for greater depth of imaging^44^.

The use of trehalose in combination with DMSO for cryopreservation was previously reported by studies in adult and fetal-like pancreatic islets^25^ and hydrogel-encapsulated islets^26^, both adding trehalose simultaneously with DMSO. Here, we maximised the protective effect of trehalose by optimising incubation times. Although we were not able to quantify the diffusion rates of trehalose itself (lack of suitable labelling and imaging modalities), the beneficial effects of trehalose were observed with 6 h pre-incubation, which correlated with the outcomes of the diffusion studies using Hoechst 33342. Prolonged pre-incubation with trehalose has been reported to improve DMSO-based cryopreservation in single cell models, to allow time for passive diffusion to the intracellular space^45,46^, but this is the first reported study to use it for enhanced diffusion into multicellular aggregates. The size of islets is expected to have an impact of the efficacy of the cryopreservation protocol. Data from the diffusion kinetics experiments suggest that the optimal pre-incubation time will be greater with increasing diameter. A given incubation time will thus only be optimal for a subset of islets in a heterogeneous batch. While sorting islets based on size is unlikely to be a feasible approach, determining the average islet size and adjusting the incubation time accordingly could help account for inter-donor variability in size distribution. Precise estimation of the relationship between islet size and incubation time should be investigated in future studies.

Addition of trehalose in the DT6h protocol improved post-thaw viability. Several hypotheses have been proposed to explain the cryoprotective effect of trehalose: stabilising proteins and membranes by water replacement^47^ or by their preferential hydration^48,49^, ability to vitrify and thus prevent crystal formation^50^ or antioxidative properties^51,52^. In our case, it is expected that trehalose remains primarily in the extracellular space, although some trehalose may cross the membrane by passive diffusion^53^ or during lipid-phase transition^25^. Even extracellular trehalose has been shown to deliver cryoprotective effect but further improvements of our protocol could be potentially be made through intracellular trehalose delivery^54–56^. Interestingly, 24 h after thawing, islets consistently displayed bi-modal viability distribution. The viability correlated with the islet size, suggesting that there may be an advantage for larger islets. We postulate a hypothesis where the survival of each islet is dependent on a critical amount of live cell–cell contact. If such trophic signals are below a critical threshold, the islet rapidly disintegrates. However, the validity of this hypothesis needs to be investigated in further studies.

While the presence of dead cells in cryopreserved samples makes some comparisons between conditions complicated, ATP/ADP, mtDNA oxidation and cAMP assays were crucial to confirm that the energetic and intracellular signalling pathways are still intact in cryopreserved islets. Release of peptides from α-cells (glucagon) and δ-cells (SST-14) appeared dysregulated in both fresh and frozen islets, possibly due to collagenase digestion damage of the islet periphery where α- and δ-cells are located^57^. Peptides from β-cells (insulin-1 and 2, IAPP) were released by fresh islets as expected in response to elevated glucose concentrations, with DT6h-cryopreserved islets following similar fold changes. DMSO-cryopreserved islets, on the other hand, showed a decrease in fold changes for these peptides, which may be due to dysregulation or passive release of stored insulin from damaged islets. Of note, the decrease in oxidative phosphorylation detected by the transcriptomic analysis in both DMSO and DT6h islets could explain some of the changes in glucose-stimulated peptide release.

Both cryopreserved groups exhibited gene expression profiles expected in cells undergoing stress: upregulation of cell death and apoptotic genes, shift away from oxidative phosphorylation and increase in pro-inflammatory markers. There is increasing evidence that metabolic stress in diabetes leads to the loss of β-cell differentiation^58^. Together with changes in cell–cell interactions, this could explain the reason for upregulation of the ‘epithelial-mesenchymal transition’ gene set in both DMSO and DT6h groups. Overall, the transcriptome profiles of DMSO and DT6h were very similar and therefore, the effect of DT6h is unlikely to be mediated through large-scale changes in transcriptional programmes. Detected differences included lower expression of inflammatory, proliferative and apoptotic genes in the DT6h group. DMSO also showed significant downregulation of the ‘cholesterol homeostasis’ gene set compared to fresh group, while DT6h had closer profile to fresh islets for these genes. This is in line with higher gene expression of cholesterol precursor (*Fdps*), enzymes involved in cholesterol biosynthesis (*Lss*, *Msmo1*) and regulators of cholesterol concentration (*Pcsk9*, *Insig1*) in DT6h compared to DMSO group. Interestingly, cholesterol is known to have a membrane stabilising effect and has been shown to improve membrane integrity after cryopreservation of sperm^59^. Cryopreservation inevitably results in membrane damage and replenishment of fatty acids and cholesterol may indicate ongoing membrane-rebuilding processes. It appears that DMSO-cryopreserved islets show significant disruption in these processes and addition of trehalose partially prevents the downregulation in the genes involved, although this must be further investigated.

In vivo evaluation of DMSO- and DT6h-cryopreserved islets revealed suboptimal performance of both groups. The likely reason is that the functional islet mass transplanted was not sufficient to consistently reverse hyperglycaemia. This is consistent with preliminary experiments where 300–400 fresh islets per animal were not sufficient to reliably reverse diabetes in all experimental animals. Cryopreservation decreased the viable islet mass by up to 50%, reducing the likelihood of achieving normoglycaemia using 800 frozen islets. Further studies using higher doses of frozen islets or their combination with fresh islets are needed to further compare the in vivo efficacy of these two cryopreservation methods. Nevertheless, the observed reduction in glucose levels upon transplantation in most animals and achievement of normoglycaemia in some confirms that frozen islets are capable of producing insulin in vivo.

The result of the pilot human islet cryopreservation experiment showed that the DT6h cryopreservation protocol, developed using mouse islets, had similar efficacy compared to DMSO alone for cryopreservation of human islets. Adaptation of mouse cryopreservation protocol to human islets will thus require further optimisation. Human islets differ significantly from mouse islets in many aspects. The majority of human islets are small^14^, with the proportion of β-cells lower in smaller islets. Moreover, β-cells populate the central part of mouse islets, whereas they are more evenly distributed both in the centre and in the periphery in human islets^60^. Shorter incubation times may thus be necessary for trehalose and DMSO to reduce cryoprotectant toxicity to β-cells. Other variations in the protocol that should be explored include the concentrations of DMSO and trehalose or temperature of the pre-incubation.

To conclude, by investigating diffusion properties of molecules in aqueous solutions into the core of multicellular aggregates, we established the basis for design of cryopreservation protocols utilising non-penetrating cryoprotectants or incubations with therapeutic agents aimed to pre-condition islets before transplantation. The methods to study diffusion and the diffusion rates identified here can be utilised in other multicellular aggregate models, such as healthy and cancer organoids and bioengineered tissues. The optimised cryopreservation method for islets, developed here using this approach, provided improvement particularly in viability, mitochondrial health and peptide release from β-cells. By combining these better quality cryopreserved pancreatic islets with fresh islets, we could reduce the direct need for this limited resource and make transplantation accessible for more patients.

## Methods

### Animals

All procedures were performed under project licences (PPL numbers 70/8702 and 80/2638) approved by the United Kingdom Home Office under the Animal (Scientific Procedures) Act 1986 in accordance with relevant guidelines and regulations (including ARRIVE guidelines). Male C57BL/6JAX mice were purchased from Charles River, UK and subsequently used for isolation of mouse pancreatic islets at the age of 5–12 weeks. Fully immunodeficient NSG (NOD.Cg-Prkdc^scid^IL2rg^tm1Wjl^/SzJ, NOD scid gamma) mice, which lack B, T and NK lymphocytes^61^ were bred in-house, litters weaned at three weeks of age and used for experiments between 6 and 13 weeks of age. All animals were housed and maintained in University Biomedical Services, University of Cambridge facility under specific-pathogen-free conditions. Mice were maintained following standard husbandry procedures in the animal facility, with ad libitum access to water and dried food pellets unless otherwise specified during experimental procedure.

### Islet isolation and culture

C57BL/6JAX mice were sacrificed by neck dislocation. Laparotomy was performed and two clamps were placed on the bowel, one on each side of the ampulla to prevent leaking of the perfusate into the bowel. 2.5 mL solution of collagenase XI (Sigma Life Science, 1000 U/mL in HBSS medium, Gibco) was slowly injected into the pancreas via the bile duct (which joins the pancreatic duct), starting from the gall bladder using a 30 G bevelled needle. The pancreas was then carefully excised, placed into a 50 mL Falcon tube containing another 2 mL of collagenase XI solution and kept on ice until pancreases from all animals were retrieved. Tubes were incubated at 37 °C in a water bath and shaken every 5 min until the tissue was digested into a fine suspension (typically 12–18 min). Digestion was then terminated by adding 25 mL of ice-cold solution of HBSS supplemented with 1 mM CaCl_2_ into each tube and centrifuging at 300*g* for 1 min at 4 °C. Two more washes with HBSS + CaCl_2_ were performed and the digest in each tube was then resuspended in 10 mL Histopaque 1077 Hybri Max™ (Sigma Aldrich) at room temperature. 5 mL room temperature RPMI medium (Gibco) was carefully layered on top of the Histopaque and tubes were centrifuged at 800*g* for 15 min at 20 °C without brake. The islet-enriched layer was collected on the interface of Histopaque and RPMI into a fresh tube, topped up with RPMI and centrifuged at 300*g* for 3 min. The pellet was transferred into a 100 mm Petri dish. Full purification was achieved by manual handpicking of islets under inverted microscope. A single pancreas yielded approximately 150 islets. These were subsequently cultured in RPMI (Gibco) + 10% fetal calf serum (FCS, Sigma Life Sciences) + 1% Penicillin/Streptomycin (Gibco) at 37 °C and 5% CO_2_ in humidified atmosphere for 1–7 days before being used for further experiments. Islets were checked for viability (> 95%) prior to the start of any experiment. Exact mouse islet counts in each experiment were determined by manual counting.

### Diffusion studies

Fresh mouse pancreatic islets were pre-stained with 400 μg/mL Hoechst 33342 nucleic acid stain (ThermoFisher) under different conditions (variations in temperature, DMSO concentration and *g* force). At the end of the incubation time, islets were washed in a Petri dish with 5 mL PBS and handpicked with minimal volume of medium (~ 10 μL) into a Peel-A-Way embedding conical mould (Polysciences, Inc.). 60–80 μL of Optimal Cutting Temperature Compound (OCT) was carefully mixed with the handpicked islets at the bottom of the mould to avoid bubbles and the drop was frozen on dry ice. Once rigid, the block was covered with more OCT, frozen on dry ice and stored at − 80 °C until sectioning. Cryo-sectioning of temperature-equilibrated OCT blocks was performed using Hacker Bright OTF5000 cryostat (Hacker Instruments and Industries Inc.) at a thickness of 11 μm. Sections were transferred to a room temperature microscope slide and frozen immediately on dry ice to avoid dehydration and further diffusion of molecules. Slides were kept on dry ice and were only removed during imaging. Prior to imaging, coverslips were mounted onto the slides using a dry mount method with double-sided tape. Wet mounting was not suitable since it allowed movement of dye molecules across the samples. Imaging was performed using the Leica Sp5 (Leica Microsystems) inverted confocal microscope as described below. Bright field and fluorescent images of 3–10 islet sections were taken for each experimental condition. Only round islets were included in the analysis as the image analysis method was not suitable to analyse irregularly shaped or elliptical islets. Image analysis was done in ImageJ plugin Concentric Circles (Inner Radius = 5.0, Outer Radius = fitted to outer edge of islet, number of Circles: calculated to be 5 px apart).

### Imaging

Wide-field images were collected using the Olympus IX81 inverted fluorescence microscope (Olympus) equipped with FITC, TRITC and DAPI filter set. Laser scanning confocal microscopy was performed using an inverted Leica Sp5 (Leica Microsystems). Detection band widths were set for each fluorochrome according to its spectral properties. Collection of images and simple pre-processing steps were performed using the LAS X software (Leica Microsystems). Multiphoton images were taken using the TriM Scope II (LaVision BioTec) upright 2-photon scanning fluorescence microscope, using appropriate set of wide bandpass emission filters. For light-sheet microscopy, pancreatic islets stained with combination of fluorescent dyes were embedded in 1% low melting agarose and drawn into a glass capillary. Once the agarose solidified, the capillary was inserted into the holder of the microscope and imaged using the Zeiss Lightsheet Z.1 (Zeiss). The excitation/emission wavelengths were 405/415 for Hoechst 33342, 488/498 for FDA and 561/571 for PI.

### Islet cryopreservation

Islets were handpicked into 1.8 mL cryovials and cryoprotectant was added according to the experimental protocol. All cryoprotectant solutions were prepared in RPMI (Gibco) + 10% FCS. Standard cryopreservation protocol (DMSO) consisted of three additions of DMSO over 45 min to gradually increase its concentration (5 min in 0.66 M DMSO at room temperature, 25 min in 1 M DMSO at room temperature and 15 min in 2 M DMSO at 4 °C). In DT6h and DT6h + MitoQ conditions, islets were pre-incubated for a specified time with 200 mM trehalose (D-( +)-trehalose dihydrate, Sigma Life Sciences) or 200 mM trehalose + 100 nM MitoQ, respectively, before the stepwise addition of DMSO described above. Freezing was performed using the CRF-1 controlled rate freezer (Grant) set to a following programme: freezing from 4 to − 7.4 °C at the rate of − 2 °C/min, holding at − 7.4 °C for 15 min, freezing to − 40 °C at the rate of − 0.3 °C/min. Vials were then plunged into liquid nitrogen and stored in the liquid phase until thawing. To thaw, samples were removed from liquid nitrogen and rapid thawing in a 37 °C water bath was performed until the last ice crystal was visible. Dilution of the cryoprotectant with culture medium (RPMI + 10% FCS) was performed using the slow step dilution protocol described previously^31^, achieving 10× dilution of the cryoprotectant. Islets were handpicked into a Petri dish containing culture medium and either used immediately or cultured for 24 h. Preparation of islets from a group of animals on the same occasion was considered as one isolation and treated as a biological replicate.

### Viability, size and yield assessment

Fresh or frozen/thawed islets were handpicked and placed in 200 μL of PBS. Islets were stained with 5 μg/mL propidium iodide (PI, Sigma-Aldrich) and 5 μg/mL fluorescein diacetate (FDA, Sigma-Aldrich) for 15 min protected from light. Stained samples were diluted with 2 mL PBS, handpicked onto a glass cover slip, covered by a second cover slip and imaged immediately by Olympus IX81 microscope with constant imaging parameters for all samples. Quantitative analysis of the images was performed using ImageJ. Manual threshold (same for all images) was applied to select the positive area in the appropriate colour channel and viability was determined as a ratio of FDA positive area and total (FDA + PI) positive area. To calculate post-thaw islet yield, number of islets recovered post-thaw was divided by number of islets prior to freezing. For size measurements, pre-freeze or thawed islets (immediately or 24 h post-thaw) were transferred into a Petri dish and bright field images of all islets were taken at 4× magnification. Diameter of each islet was manually measured using ImageJ.

### ATP/ADP ratio assay

50 pancreatic islets per condition were handpicked into a 1.5 mL Eppendorf tube and adenonucleotides were extracted by adding 1 mL of ice-cold perchloric acid (3% v/v HClO_4_, 2 mM Na_2_EDTA, 0.5% Triton X-100) followed by vortexing. Extracted samples were stored at − 80 °C until ATP and ADP quantification using luciferase-based assay described by Strehler^62^. Briefly, ATP and ADP standard curves were prepared by serial dilutions in perchloric acid from 20 mM stock solutions of ATP and ADP sodium salts (Sigma). 400 μL of each sample was pH-neutralised using ice-cold KOH solution (2 M KOH, 2 mM Na_2_EDTA, 50 mM MOPS) until white KClO_4_ precipitate formed. Samples were centrifuged (17,000*g*, 1 min, 4 °C) and supernatant used for further measurements. 250 μL of sample supernatant was prepared for quantification of ADP by degradation of endogenous ATP by incubating the supernatant with 250 μL ATP sulfurylase assay buffer (20 mM Na_2_MoO_4_, 5 mM GMP, 0.2 U ATP sulfurylase (New England Biolabs), in Tris–HCl buffer (100 mM Tris–HCl, 10 mM MgCl_2_ (pH 8.0))) for 30 min at 30 °C while shaking, followed by heating to 100 °C for 5 min and cooling on ice. 100 μL of standards, one 100 μL sample for ATP measurement and two 200 μL samples for ADP measurement were mixed in luminometer tubes with 400 μL Tris–acetate buffer (100 mM Tris, 2 mM Na_2_EDTA, 50 mM MgCl_2_, pH adjusted to 7.75 with glacial acetic acid). To one of the ADP measurement samples, 10 μL pyruvate kinase solution (100 mM PEP, 6 U pyruvate kinase suspension (Sigma)) was added and incubated for 30 min at 25 °C in the dark to convert ADP to ATP. The other ADP measurement sample remained unconverted and served as a blank. 100 μL Luciferase/Luciferin Solution (7.5 mM DTT, 0.4 mg/mL BSA, 1.92 μg luciferase/mL (Sigma), 120 μM d-luciferin (Sigma), in Tris-acetate buffer (25% v/v glycerol)) was auto-injected and luciferase luminescence measured using the Berthold AutoLumat Plus luminometer three times for 1 min and the data quantified against standard curves using MS Excel.

### mtDNA oxidation assay

Oxidation of mtDNA was assayed by quantitative polymerase chain reaction (PCR) using two targets: a short target (≥ 200 bp), to control for the mtDNA copy number, and a long target (~ 10 kbp), for which reduced amplification corresponds to oxidative DNA damage^35,36^. DNA was isolated from 50 fresh or thawed pancreatic islets using GenElute™ Mammalian Genomic DNA Miniprep Kit (Sigma-Aldrich) according to manufacturer’s instructions and DNA stored at − 20 °C until the assay. Prior to the assay, DNA concentration in the thawed samples was measured using NanoDropTM 8000 Spectrophotometer (ThermoFisher Scientific) and samples were diluted to 3 ng/mL. The PCR mastermix contained 200 μM dNTPs (TμaKaRa), 200 pM forward primer (Sigma, 5′-GCC AGC CTG ACC CAT AGC CAT AAT-3′), 200 pM reverse primer (Sigma, 5′-GCC GGC TGC GTA TTC TAC GTT A-3′ for short target, 5′-GAG AGA TTT TAT GGG TGT AAT GCG G-3′ for long target), 100 ng/mL BSA (New England Biolabs) and 1 mM Mg (OAc)_2_ (TaKaRa) in buffer (TaKaRa). Reaction was performed in PCR tubes and each reaction consisted of 5 μL (15 ng) of DNA template from samples, 35 μL of PCR mastermix and 1 U of LA Taq polymerase (TaKaRa). Reactions were performed in duplicates, including non-template control and linear amplification control. Reactions were then performed using 22 cycles for short target and 25 cycles for long target. PCR products were quantified using the Quant-iT™ PicoGreen™ dsDNA Assay Kit (ThermoFisher Scientific) according to manufacturer’s instructions and the size of the PCR product was checked on a 0.8% agarose gel. For the analysis in Excel, all samples were corrected to the non-template control and normalised to amplification of the short target.

### cAMP assay

Fresh or thawed pancreatic islets (150–200 islets/condition) were dissociated into single cells using 5 min incubation with 0.05% trypsin + 0.53 mM EDTA (Sigma-Aldrich) and mechanical disruption with p1000 pipette. Dissociation was stopped by addition of RPMI + 10% FCS and cells were counted using haemocytometer. Measurements of cAMP were performed using LANCE^®^ cAMP Detection Kit (PerkinElmer) in accordance with the manufacturer’s instructions. Assay was run in duplicates or triplicates, using 4000 cells per well in 384-well OptiPlate (PerkinElmer). Forskolin (Sigma Aldrich), GLP-1(7–26)amide (Alta Biosciences), cAMP standard or DMSO control were diluted in stimulation buffer (PBS supplemented with 0.1% BSA (Sigma-Aldrich, A2153) and 0.5 mM of the non-specific inhibitor of cAMP and cGMP phosphodiesterases (PDEs) 3-Isobutyl-1-methylxanthine (IBMX) (Sigma-Aldrich)) to the appropriate concentration (final concentration of 10^–4^ M forskolin, 10^–5^ or 10^–6^ M GLP-1(7–36)amide in assay). Measurements were taken using a Mithras LB 940 microplate reader (Berthold Technology); excitation 340 nm and emission 665 nm.

### Peptidomics

Fresh or thawed pancreatic islets were thoroughly washed from any leftover cryoprotectant (to avoid an effect of remaining trehalose on the insulin secretion). Islets were acclimatised for 30 min in pre-warmed 3.3 mM glucose Krebs–Ringer Bicarbonate Buffer (KRB (mM): 137 NaCl, 4.7 KCl, 1.2 KH_2_PO_4_, 1.2 MgSO_4_·7H_2_O, 2.5 CaCl_2_·2H_2_O, 25 NaHCO_3_). Islets were washed once in fresh 3.3 mM KRB before assay. Each condition was tested in triplicates. 10 islets were handpicked into 1.5 mL Protein LoBind tubes (Eppendorf) and incubated with 500 μL of 3.3 mM KRB for 1 h in a humidified chamber at 37 °C. Then, tubes were mixed and spun in a microcentrifuge (300*g* for 30 s) and all supernatant transferred into a new Protein LoBind tube and stored at − 80 °C. Next, same islets were incubated with 500 μL of 16.7 mM glucose KRB for 1 h at 37 °C while shaking, then mixed, centrifuged and the supernatant taken out into a new Protein LoBind tube. Supernatants were stored at − 80 °C until analysis by liquid chromatography tandem mass spectrometry (LC–MS/MS). Prior to protein extraction, islet supernatants were thawed on ice and acidified with 50 μL 1% formic acid in H_2_O (v/v). A stable isotope-labelled internal standard (IS) for glucagon was spiked into the samples for a final concentration of 20 ng/mL. Samples were loaded onto a HLB Prime μElution plate (Waters) for solid phase extraction (SPE). 200 μL 0.1% formic acid in H_2_O was used to wash the wells prior to a wash with 200 μL 1% acetic acid and 5% methanol in H_2_O (v/v/v). Peptides were eluted into a Protein LoBind 96 well plate (Eppendorf) with 2 separate elutions of 30 μL 10% acetic acid, 10% methanol. 40 μL 0.1% formic acid in H_2_O was added to all samples to prior to LC–MS/MS analysis to reduce the organic solvent composition of the extract. A H-Class Aquity (Waters) LC system coupled to a Xevo TQ-XS triple quadrupole mass spectrometer (Waters) was used to quantify islet derived peptide hormones in supernatants. 40 μL of sample was loaded onto a 2.1 × 50 mm 1.8 μm particle HSS T3 Aquity column at 60 °C flowing at 350 μL/min with gradient starting conditions set to 90% solvent A (0.1% formic acid in H_2_O) and 10% solvent B (0.1% formic acid in acetonitrile (Pierce) (v/v)) for 30 s. The percentage of solvent B was gradually increased to 50% over 7.5 min, and the column was washed with 90% solvent B for 2 min before returning to initial conditions with a total run time of 10 min. LC–MS/MS analysis was performed using positive electrospray ionisation (ESI) with a spray voltage of 3 kV, desolvation temperature 600 °C, gas flow rate 1000 L/h and cone voltage of 40 V. Table 1 details the precursor and product ions for each of the islet hormones analysed in addition to collision energies and dwell times. Peptides were quantified by integrating the corresponding peaks using TargetLynx v4.2 software (Waters). Raw peak area values are reported as a measure of peptide abundance in the sample apart from glucagon which is normalised to its internal standard and presented as peak area ratio.

**Table 1:** Parameters of LC–MS/MS analysis.

Peptide	Precursor *(m/z)*	Product *(m/z)*	Collision energy	Dwell time (ms)
Insulin-1	967.8	331.2	40	0.025
Insulin-2	1159.7	334.1	40	0.025
IAPP	980.5	958.2	20	0.025
Glucagon	871.5	1040.2	27	0.025
Glucagon IS	877.1	1047.2	27	0.025
Somatostatin-14	546.6	726.3	15	0.025

Precursor and precursor ions along with collision energies and dwell time used to monitor each peptide during LC–MS/MS analysis.

### RNA sequencing

Isolated pancreatic islets were cultured for 24 h and split into three groups: fresh, DMSO and DT6h. In the fresh group, 200 islets were centrifuged (300*g*, 1 min) and the pellet resuspended in 100 μL RNAlater solution (LifeTechnologies) and then stored at − 20 °C until RNA isolation. In the DMSO and DT6h groups, 200 islets were cryopreserved using either the standard DMSO or DT6h cryopreservation protocol described above. After thawing, islets were cultured for 24 h, after which the islets from each group were centrifuged (300*g*, 1 min), resuspended in 100 μL RNAlater solution (LifeTechnologies) and stored at -20 °C until RNA isolation. Total cellular RNA was isolated using a Qiagen RNeasy Plus Micro kit (Qiagen). Briefly, 0.5 mL cold PBS was added to each pancreatic islet sample, gently mixed by inversion and the islets spun down at 600*g* for 5 min in a microfuge. The pellet was resuspended and lysed in 350 μL RLT Plus buffer. The resulting lysate was then passed over a QIA Shredder column (Qiagen) to optimise homogenisation of the resulting lysate. Subsequent steps in RNA isolation were according to the manufacturer’s instructions. Total RNA was eluted in a volume of 14 μL RNAse-free water, snap-frozen on dry ice and stored at − 80 °C. RNA quality and quantity were measured on an Agilent 4200 TapeStation system and 50 ng used for subsequent library construction. Ribosomal RNA was removed using the QIA FastSelect RNA Removal kit (Qiagen), with a 10 min incubation at 95 °C, to produce insert sizes in the 150–250 bp range. Strand-specific libraries were then constructed using the QIAseq Stranded Total RNA Library kit (Qiagen) according to the manufacturer's instructions. Library quality was assessed on an Agilent 4200 TapeStation system and quantified by qPCR using the QIAseq Library Quant Assay kit (Qiagen). Libraries were pooled to an equimolar ratio and single-end (50 bp reads) sequencing carried out on a HiSeq 4000 to an average depth of 26.7 million reads per library. FastQ files were demultiplexed using bcl2fastq (Illumina), trimmed using TrimGalore! and mapped to the mouse genome (GRCm38) using HiSat2. Bam files were read into SeqMonk^39^ (Babraham Institute, Cambridge) for read quantitation and library normalisation. Differentially expressed genes were identified separately by DESeq2^38^ and the Intensity Difference filter^40^ (a fold-change filter weighted for gene expression). A set of genes common to both the DESeq2 and Intensity Difference filters was used for some visualisations. For pathway analysis, Gene Set Enrichment Analysis (GSEA) was run in pre-ranked mode (GSEA-Preranked) against the Hallmark gene set collection from the Molecular Signatures Database (MSigDB) and C5 Gene Ontology: Biological Process gene sets^63^. The inverse of the raw p-value was used as a ranking metric. The direction of change in expression was given by the sign of the log fold change. Gene sets with an FDR q-value ≤ 0.05 were considered significant. Plots were produced using GraphPad Prism (version 8.4.1) or SeqMonk (version 1.45.4).

### Transplantation of islets into STZ-diabetic mice

Streptozotocin (STZ, Sigma Life Science) solution was prepared on the day of the injection by dissolving STZ powder in freshly prepared citrate buffer (0.1 M citric acid + 0.1 M sodium citrate, pH 4.2) at 10 mg/mL and kept on ice until the injections. NSG mice were weighed, anaesthesised using mixture of isoflurane and oxygen and injected with 40 mg/kg STZ intraperitoneally on 5 consecutive days using 30 G needle. In addition to their regular chow, mice were fed mash made of powdered diet with the same composition to prevent weight loss for the duration of the experiment. Mice were weighed and blood glucose was measured using iHealth Smart Gluco-Monitoring system (iHealth Labs) by tail vein bleeds before the first injection of STZ and then weekly until the end of the experiment after 6 h of fasting. Mice were considered diabetic when their fasting blood glucose reached over 13.5 mmol/L and used for transplantation when fasting blood glucose reached over 18 mmol/L. Diabetic animals were transplanted with 800 fresh or DMSO- or DT6h- cryopreserved islets resuspended in Matrigel (Corning) under the kidney capsule. Preliminary optimisation of transplanted islet dose (testing 300, 400 and 800 islets per animal) was performed in a similar manner. Animals were culled at > 20% weight loss (1 animal in the Matrigel group) or 75 days after transplantation. At the endpoint, blood was collected for serum using inferior vena cava bleed under terminal anaesthesia. To extract serum, samples were kept at 4 °C for 24 h after collection to allow for coagulation and spun in a microcentrifuge at 13,000*g* for 7 min. Clear serum was collected from the top supernatant layer and samples were stored at − 20 °C until analysis. Serum glucose was measured using a colorimetric assay on the Siemens Dimension EXL autoanalyser. All reagents and calibrators were supplied by Siemens.

### Cryopreservation of human pancreatic islets

Pancreatic islets were isolated from pancreases of human deceased organs donors by SNBTS Islet Cell Laboratory in Edinburgh (ethical approval REC Ref: 16/WM/0093). These clinically isolated islets were declined for transplantation due to low purity, viability or yield and subsequently offered for research use. They were shipped in CMRL 1066 Supplemented culture media (Corning) at room temperature and further processed upon arrival. The purity and viability of the received islets was validated upon receipt in our laboratory using dithizone staining and fluorescein diacetate/propidium iodide staining. Clinical and islet isolation information about human islet preparations can be found in Supplementary Table 1. Islets were cryopreserved and viability was tested using the same protocol as for mouse islets, CMRL 1066 was used instead of RPMI, islet counts were determined by manual handpicking.

### Statistical analysis

Sample sizes for in vitro and in vivo experiments were computed using the G*Power software (version 3.1) prior to the experimental work. Effect size was based on data available from a pilot experiment, power was set to 80% and α error probability to 0.05. Statistical analysis was performed using the GraphPad Prism software (version 8.4.1) using t-test, one-way or two-way ANOVA followed by Tukey’s, Sidak’s or Dunnett’s post-hoc tests as appropriate. Differences were considered statistically significant if p-value ≤ 0.05. In graphs, individual data points or mean values ± SD are shown. Correlations were evaluated using Spearman’s correlation coefficient and reported along with p-values. Results obtained from viability staining of pancreatic islets at 24 h after thawing followed bimodal distribution. Calculation of means using standard methods was used as an approximation to determine average viability of each sample. These means then followed Gaussian distribution and were analysed using standard methods described above. Animals were randomly assigned to treatment groups; littermates were used, and data was analysed blinded to the group identity.

## Supplementary Information

Supplementary Information.
