# Supplementary material for: Accelerating cryoprotectant diffusion kinetics improves cryopreservation of pancreatic islets
Source: Sci Rep. 2021 May 17;11:10418. doi: 10.1038/s41598-021-89853-6 (PMC8129116; doi:10.1038/s41598-021-89853-6)
Supplement: Supplementary file 1 — Supplementary Information. [file 41598_2021_89853_MOESM1_ESM.pdf]

Supplementary Information

Accelerating cryoprotectant diffusion kinetics improves cryopreservation of pancreatic islets

Nikola Dolezalova[1,2], Anja Gruszczyk[3], Kerry Barkan[4,5], John A. Gamble[1], Sam Galvin[6], Till Moreth[7], Kevin O'Holleran[8], Krishnaa T. Mahbubani[1], Jackie A. Higgins[1], Fiona M. Gribble[6], Frank Reimann[6], Jakub Surmacki[9], Simon Andrews[10], John J. Casey[11], Francesco Pampaloni[7], Michael P. Murphy[3,12], Graham Ladds[4], Nigel K. H. Slater[2], Kourosh Saeb-Parsy[1*]

[1] Department of Surgery, University of Cambridge, Box 202, Addenbrooke's Hospital, Hills Road, Cambridge CB2 0QQ, UK. [2]Department of Chemical Engineering and Biotechnology, University of Cambridge, Philippa Fawcett Drive, Cambridge CB3 0AS, UK. [3]MRC Mitochondrial Biology Unit, University of Cambridge, Keith Peters Building, Cambridge Biomedical Campus, Hills Rd, Cambridge CB2 0XY, UK. [4]Department of Pharmacology, University of Cambridge, Tennis Ct Rd, Cambridge CB2 1PD, UK. [5]Sosei Heptares, Steinmetz Building, Granta Park, Cambridge CB21 6DG, UK , [6]Institute of Metabolic Science, University of Cambridge, Box 289, Addenbrooke's Hospital, Cambridge CB2 0QQ, UK. [7]Buchman Institute for Molecular Life Science, Goethe University Frankfurt, Max-von-Laue-Straße 15, 60438 Frankfurt am Main, Germany. [8]Cambridge Advanced Imaging Centre, University of Cambridge, Anatomy Building, Downing Site, Cambridge CB2 3DY, UK. [9]Cavendish Laboratory, University of Cambridge, JJ Thomson Ave, Cambridge CB3 0HE, UK. [10]Bioinformatics Group, The Babraham Institute, Cambridge, CB22 3AT. [11]Department of Clinical Surgery, University of Edinburgh, Royal Infirmary of Edinburgh, Edinburgh EH16 4SA, UK. [12]Department of Medicine, University of Cambridge, Box 157, Addenbrooke's Hospital, Hills Road, Cambridge, CB2 2QQ. [*]Corresponding author; email: ks10014@cam.ac.uk

Supplementary Table 1: Clinical and islet isolation information about human islet preparations. *n/a: data not available*

Characteristic	Islet preparation		
	Donor 1	Donor 2	Donor 3
Donor demographics			
Age (years)	55	51	47
Sex (M/F)	F	M	M
BMI (kg/m^2)	27	27	29
Ethnicity	white	white	white
HbA1c	n/a	n/a	n/a
Cause of death	intracranial haemorrhage	intracranial thrombosis	intracranial – unclassified
Diabetes (Y/N)	N	N	N
Pancreas			
Source	Kirkcaldy	Hull	Newport
Warm ischaemia time (h)	7 h 2 min	2 h 12 min	1 h 3 min
Cold ischaemia time (h)	n/a	14 h 9 min	11 h 24 min
Islet handling and use			
Isolation centre	Edinburgh	Edinburgh	Edinburgh
Estimated purity (%)	90	54	49
Estimated viability	85	n/a	n/a
Total culture time (h)	70	45	70

This table was adapted from: N. J. Hart and A. C. Powers. Use of human islets to understand islet biology and diabetes: progress, challenges and suggestions. *Diabetologia*, 62(2):212–222, 2019.

Supplementary Table 2: Selected non-significant pathways from GSEA-Preranked of differences in Gene Ontology: Biological Process gene set expression levels between DT6h and DMSO-cryopreserved islets. Selected pathways with FDR > 0.05 are shown. along with normalised Enrichment Score (positive for pathways with higher expression in DT6h, negative for pathways with higher expression in DMSO).

	NES	FDR-q
**Insulin-related pathways**		
GO_CELLULAR_RESPONSE_TO_INSULIN_STIMULUS	2.0856302	0.999
GO_POSITIVE_REGULATION_OF_CELLULAR_RESPONSE_TO_INSULIN_STIMULUS	2.054333	0.999
GO_INSULIN_RECEPTOR_SIGNALING_PATHWAY	1.9114529	1
GO_INSULIN_SECRETION	1.0580891	1
GO_REGULATION_OF_CELLULAR_RESPONSE_TO_INSULIN_STIMULUS	0.9618487	1
GO_NEGATIVE_REGULATION_OF_CELLULAR_RESPONSE_TO_INSULIN_STIMULUS	0.8433451	1
GO_INSULIN_SECRETION_INVOLVED_IN_CELLULAR_RESPONSE_TO_GLUCOSE_STIMULUS	0.7125794	1
GO_REGULATION_OF_INSULIN_SECRETION	0.6884126	1
GO_POSITIVE_REGULATION_OF_INSULIN_SECRETION_INVOLVED_IN_CELLULAR_RESPONSE_TO_GLUCOSE_STIMULUS	0.616469	1
GO_POSITIVE_REGULATION_OF_INSULIN_SECRETION	-1.2167271	1
**Glucose metabolism, transport and regulation**		
GO_GLUCOSE_METABOLIC_PROCESS	1.6793224	1
GO_POSITIVE_REGULATION_OF_GLUCOSE_METABOLIC_PROCESS	1.3504944	1
GO_GLUCOSE_IMPORT	1.317331	1
GO_REGULATION_OF_GLUCOSE_METABOLIC_PROCESS	1.2233201	1
GO_REGULATION_OF_GLUCOSE_IMPORT	1.1557162	1
GO_CELLULAR_GLUCOSE_HOMEOSTASIS	1.147696	1
GO_GLUCOSE_CATABOLIC_PROCESS	1.1136378	1
GO_NEGATIVE_REGULATION_OF_GLUCOSE_TRANSMEMBRANE_TRANSPORT	0.8583012	1
GO_POSITIVE_REGULATION_OF_GLUCOSE_TRANSMEMBRANE_TRANSPORT	0.7460093	1
GO_CELLULAR_RESPONSE_TO_GLUCOSE_STARVATION	-1.7592208	1
GO_GLUCOSE_6_PHOSPHATE_METABOLIC_PROCESS	-1.1514777	1
GO_REGULATION_OF_GLUCOSE_TRANSMEMBRANE_TRANSPORT	-0.7172715	1
**Exocytosis-related pathways**		
GO_EXOCYTIC_PROCESS	1.8231285	1
GO_REGULATION_OF_EXOCYTOSIS	1.7921743	1
GO_CALCIUM_ION_REGULATED_EXOCYTOSIS	1.7872827	1
GO_REGULATION_OF_CALCIUM_ION_DEPENDENT_EXOCYTOSIS	1.6098738	1
GO_POSITIVE_REGULATION_OF_CALCIUM_ION_DEPENDENT_EXOCYTOSIS	1.4485811	1
GO_VESICLE_DOCKING_INVOLVED_IN_EXOCYTOSIS	1.3660048	1
GO_POSITIVE_REGULATION_OF_EXOCYTOSIS	0.91602623	1
GO_NEGATIVE_REGULATION_OF_EXOCYTOSIS	-1.4453918	1

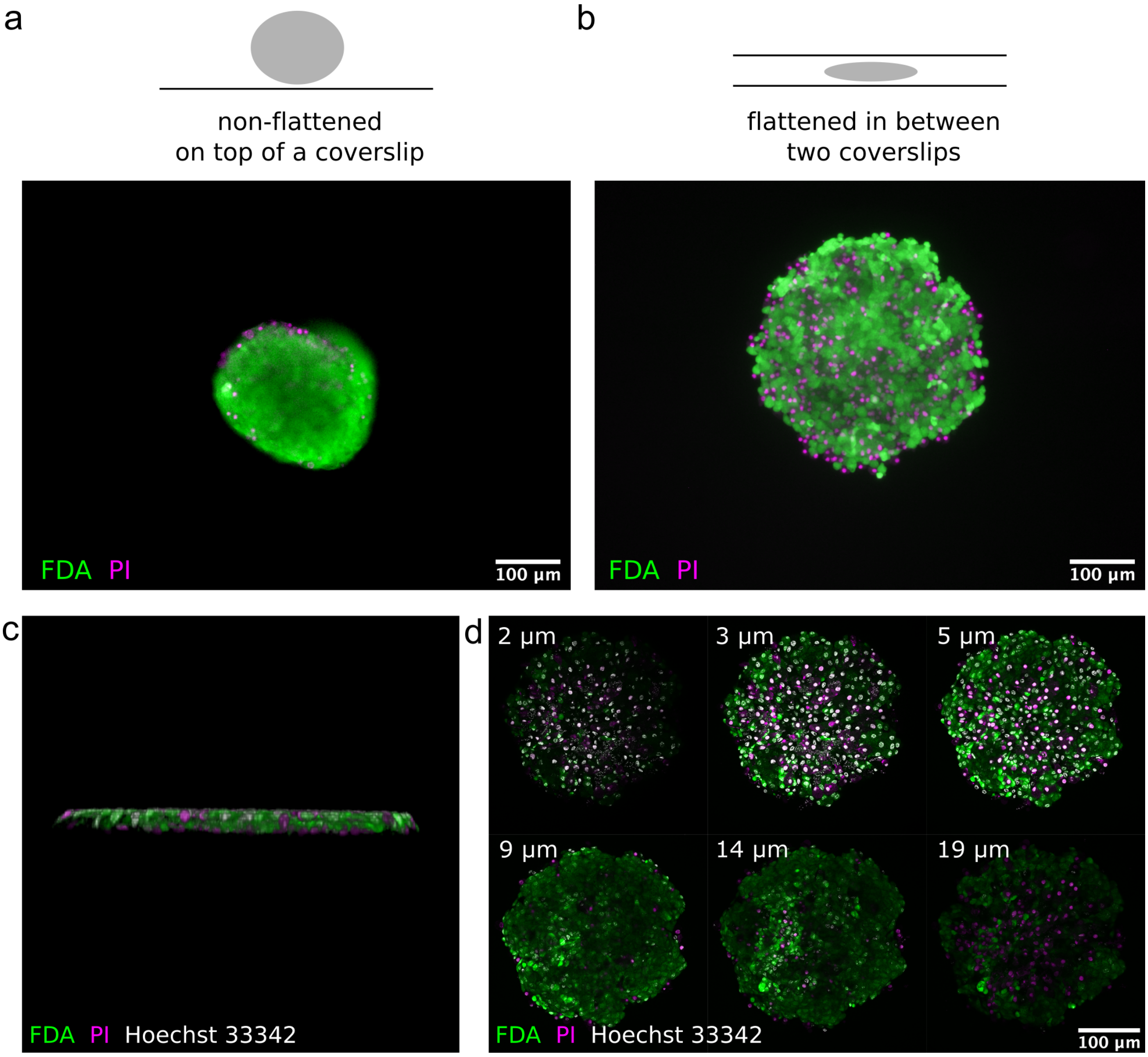

Supplementary Figure 1: **Viability staining of pancreatic islets.** Using wide-field fluorescence microscopy, it is not possible to image all cells in non-flattened pancreatic islets. By placing islets in between two coverslips, islets are flattened to approximately 20 μm thickness (corresponding to 2–3 layers of cells), which enables more accurate monitoring of islet viability. **(a)** Pancreatic islet stained with FDA and PI placed on top of a coverslip imaged by a wide-field fluorescence microscope. Because wide-field microscopy uses non-directed light, the visible cells are located mostly at the top outer layer of the stained pancreatic islets. **(b)** Pancreatic islet stained with FDA and PI flattened in between two coverslips imaged by a wide-field fluorescence microscope. While the information about original spatial localisation of each cell is lost, majority of cells in the thinner disc are in the plane of focus and it is thus possible to detect viability of most cells. **(c)** Side view of 3D-reconstructed flattened pancreatic islet imaged on a confocal microscope (FDA/PI/H33342 staining).
3D reconstruction performed using the 3D Viewer plugin in Fiji. **(d)** Lateral cross-sections of the 3D image data of the flattened pancreatic islet in between two coverslips imaged on a confocal microscope (FDA/PI/Hoechst 33342 staining); each slice is labelled with distance in µm from the top of the islet.

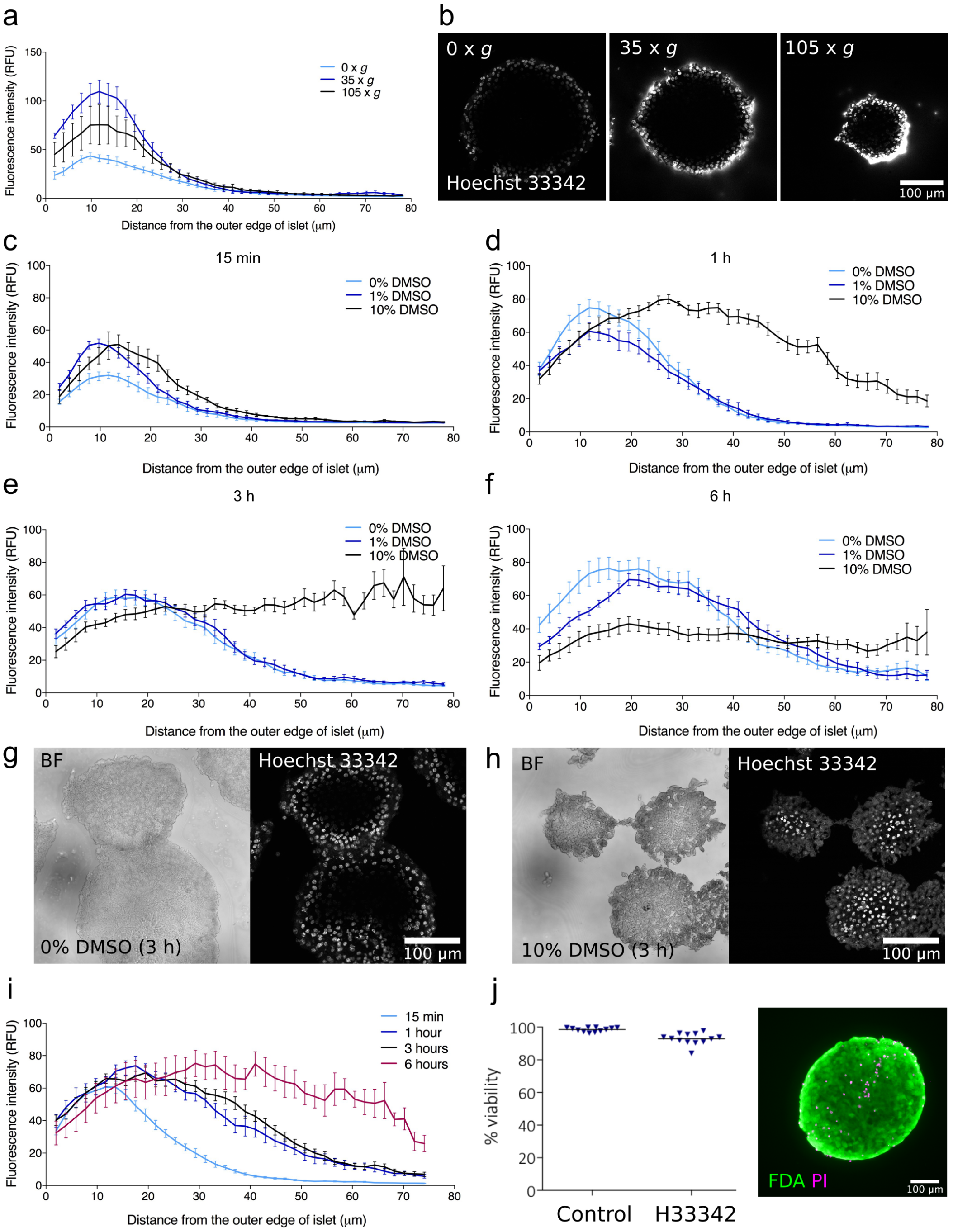

Supplementary Figure 2: Studies of diffusion of nuclear dye Hoechst 33342 into pancreatic islets. **(a)** Staining profiles of islets incubated with Hoechst 33342 for 15 min at varying hydrostatic pressures (n = 6). **(b)** Fluorescence images of islets incubated with Hoechst 33342 for 15 min at 0 × *g* (left), 35 × *g* (centre) and 105 x *g* (right). **(c)–(f)** Staining profiles of islets incubated with Hoechst 33342 in the presence of 0%, 1% or 10% DMSO for 15 min (c), 1 h (d), 4 h (e) or 6 h (f). **(g)** Bright-field and Hoechst 33342 fluorescence images of islets incubated with 0 % DMSO for 3 h. **(h)** Bright-field and Hoechst 33342 fluorescence images of islets incubated with 10 % DMSO for 3 h. **(i)** Staining profiles of pancreatic islets incubated Hoechst 33342 for 15 min, 1 h, 3 h or 6 h at 37 °C (n = 7–15). **(j)** Viability of pancreatic islets incubated for 6 h at 37 °C in culture medium (Control) or Hoechst 33342 staining solution. On the right, a representative image of a pancreatic islet incubated with Hoechst 33342 for 6 h, stained for viability (using FDA and PI) at the end of the incubation and imaged by wide-field fluorescent microscope. All plots were created using GraphPad Prism (version 8.4.1, https://www.graphpad.com). *BF = bright field; H33342 = Hoechst 33342.*

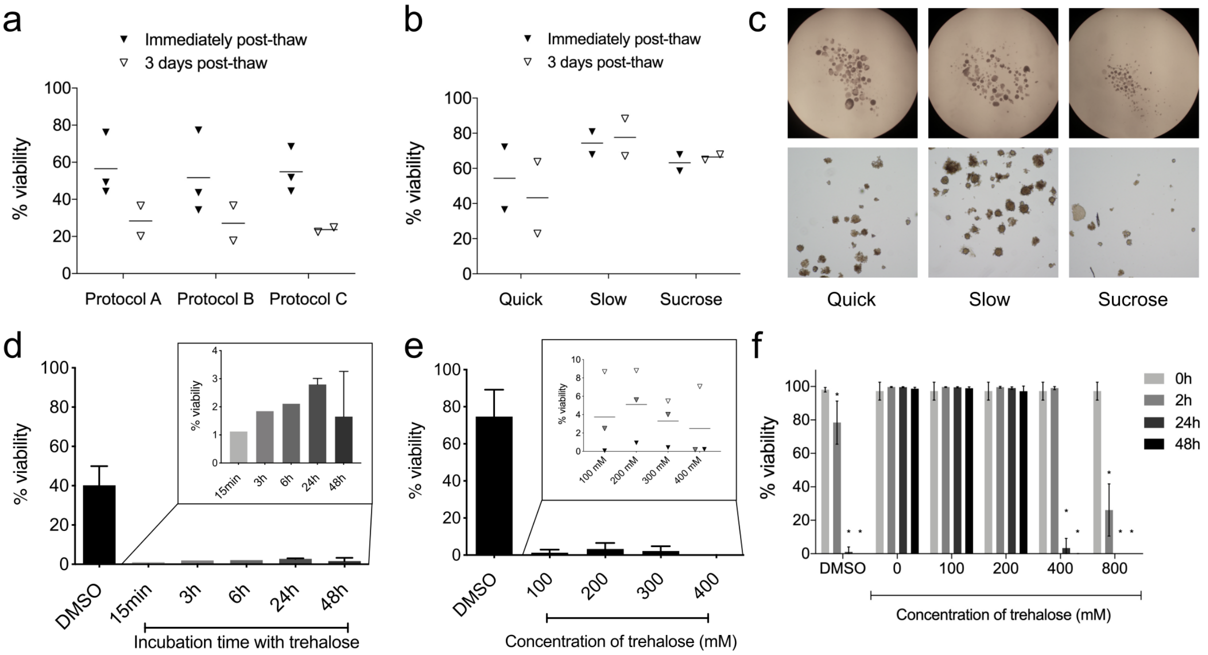

Supplementary Figure 3: Optimisation of the general parameters of the cryopreservation protocol. (a) Comparison of three DMSO addition protocols for pancreatic islets. Post-thaw viability immediately after thawing and 3 days after thawing is shown. Protocol A: step-wise addition of DMSO over 30 min at room temperature, followed by 15 min of equilibration on ice (suggested by Rajotte). Protocol B: single step addition of DMSO with 15 min equilibration on ice. Protocol C: single-step addition with no equilibration before freezing. Each triangle represents the average viability from one experimental sample calculated from 5–14 islets; horizontal line represents the mean (n = 2–3) **(b)** Comparison of three thawing protocols for pancreatic islets. Post-thaw viability of islets immediately after thawing and 3 days after thawing is shown. Quick: 10x dilution of DMSO in a single step. Slow: step-wise dilution of DMSO using culture medium over 24 min until 10x dilution is reached (suggested by Lakey *et al.*). Sucrose: at time 0, DMSO is exchanged for 0.75 M sucrose and incubated on ice for 30 min, followed by step-wise dilution of sucrose (suggested by Rajotte and Mazur). Each triangle represents the average viability of one experimental sample calculated from 6–14 islets; horizontal line represents the mean (n = 2). **(c)** Bright field images of mouse pancreatic islets thawed using three different protocols. Top row shows all islets recovered after thawing; bottom row show the same islets under 4x magnification. **(d)** Post-thaw viability of mouse pancreatic islets cryopreserved with DMSO (no extra incubation) or 200 mM trehalose (15 min, 3 h, 6 h, 24 h or 48 h incubation prior to freezing). Viabilities of the trehalose-cryopreserved islets are shown in the zoomed graph on the right. Bars represent mean ± SD, n = 1–2. **(e)** Post-thaw viability of mouse pancreatic islets cryopreserved with DMSO or different trehalose concentrations (100–400 mM). Viabilities of the individual replicates of trehalose-cryopreserved islets are shown in the insert with matched symbols for each replicate; n = 3. **(f)** Viability of mouse pancreatic islets after incubation with 2 M DMSO or different concentrations of trehalose (0–800 mM; mean ± SD of one experiment shown, viability of each experimental condition calculated as an average of 1–12 islets). Differences between each group and control group (Control or 0 mM trehalose) were tested by two-way ANOVA followed by Dunnett's test. Significant differences ($p \leq 0.05$) are marked by an asterisk. All plots were created using GraphPad Prism (version 8.4.1, https://www.graphpad.com).

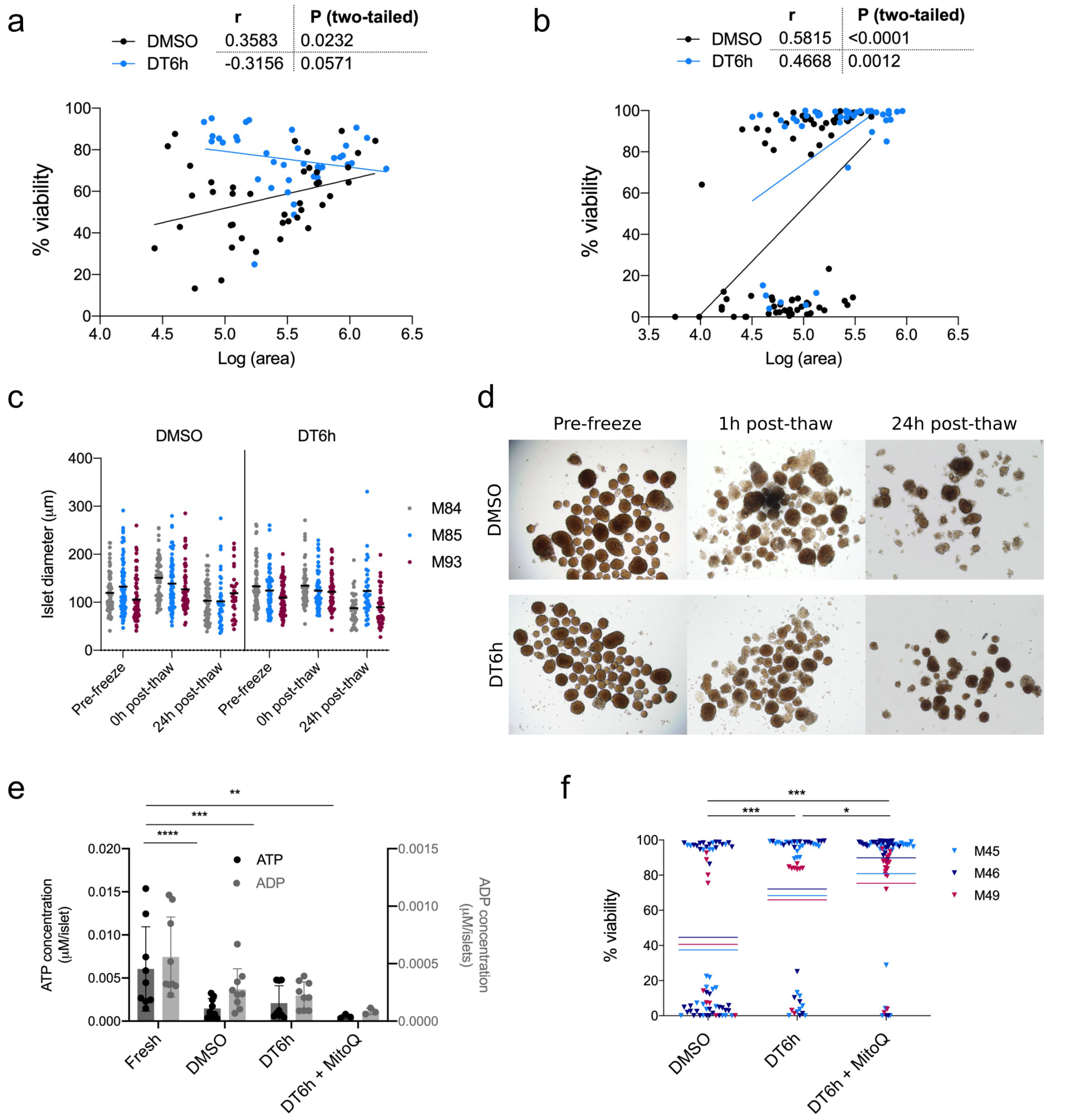

Supplementary Figure 4: Morphological, metabolic and functional assessment of the new DT6h protocol. (a–b) Correlation of viability and size (measured as the imaged area in pixels) in the DMSO (black) and DT6h (blue) groups immediate post-thaw (b) and 24 h post-thaw (c). Islets from three independent experiments were included, each circle represents an individual islet. Corresponding Spearman r and p-values are shown above the figure. **(c)** Diameter of individual DMSO- and DT6h-cryopreserved islets measured pre-freeze, 0 h post-thaw and 24 h post-thaw. Circles of the same colour represent results from a single experiment. Summarised results can be found in Figure 4g. **(d)** Bright-field microscopy images of DMSO- and DT6h-cryopreserved islets before and after freezing, taken at 4x magnification. After 1 h post-thaw imaging, half of islets were taken from the sample for viability assessment, 24 h post-thaw image therefore contains only half of the original number of islets. Representative images of n = 3 experiments. **(e)** Absolute concentrations of ATP (black, left axis) and ADP (grey, right axis) measured in fresh and DMSO-, DT6h- or DT6h+MitoQ-cryopreserved islets 24 h after thawing. **(f)** Post-thaw viability of islets cryopreserved with DMSO, DT6h and DT6h + MitoQ measured 24 h after thawing. Triangles represent individual islet viabilities; triangles of same colour represent results from the same experiment. Repeated measures one-way ANOVA followed with Tukey's HSD post-hoc test was performed, statistically significant difference is marked by asterisks: * $p \leq 0.05$, ** $p \leq 0.01$, *** $p \leq 0.001$, **** $p \leq 0.0001$. All plots were created using GraphPad Prism (version 8.4.1, https://www.graphpad.com).

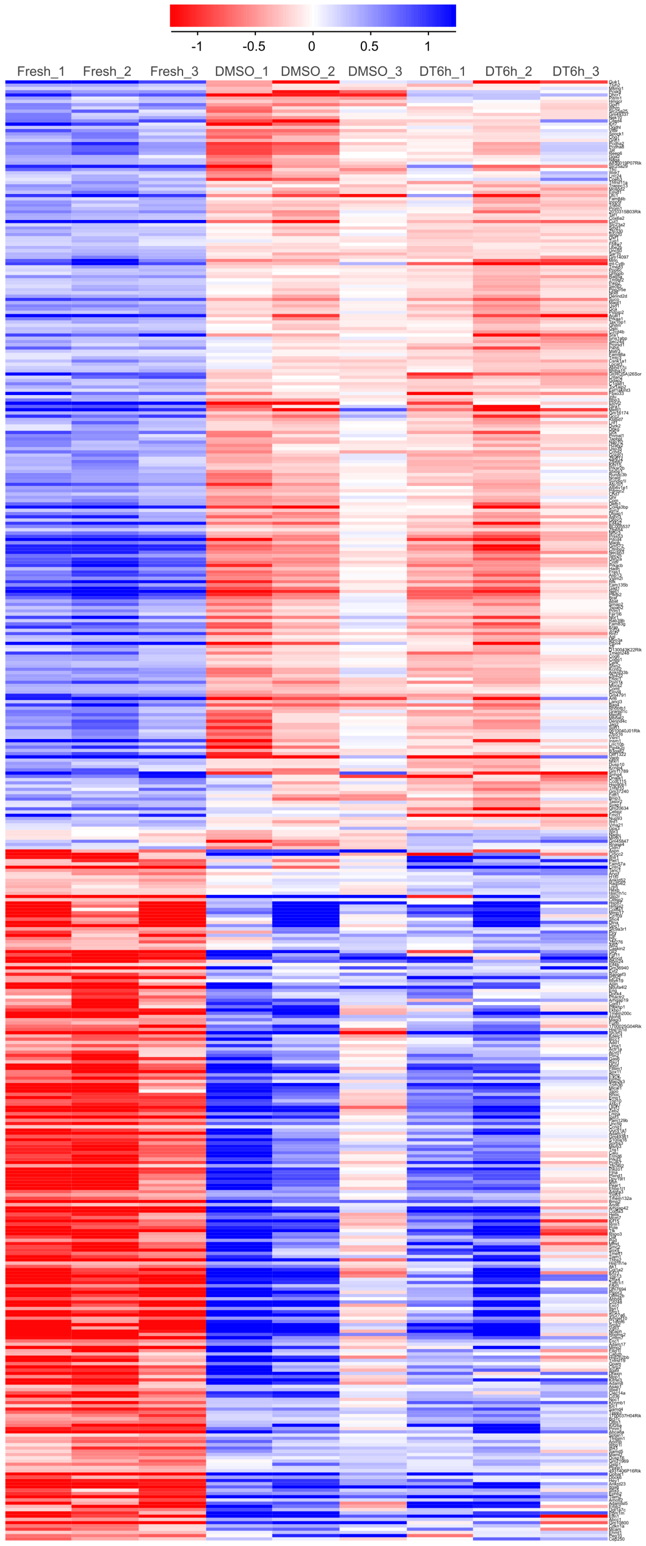

Supplementary Figure 5: Bulk RNA sequencing of fresh, DMSO- and DT6h-cryopreserved islets. **(a)** Heatmap showing the 2868 genes that are differentially expressed between the three tested conditions (assessed by DESeq2, FDR ≤ 0.05, n = 3, individual replicates shown). The heatmap was generated using SeqMonk software.

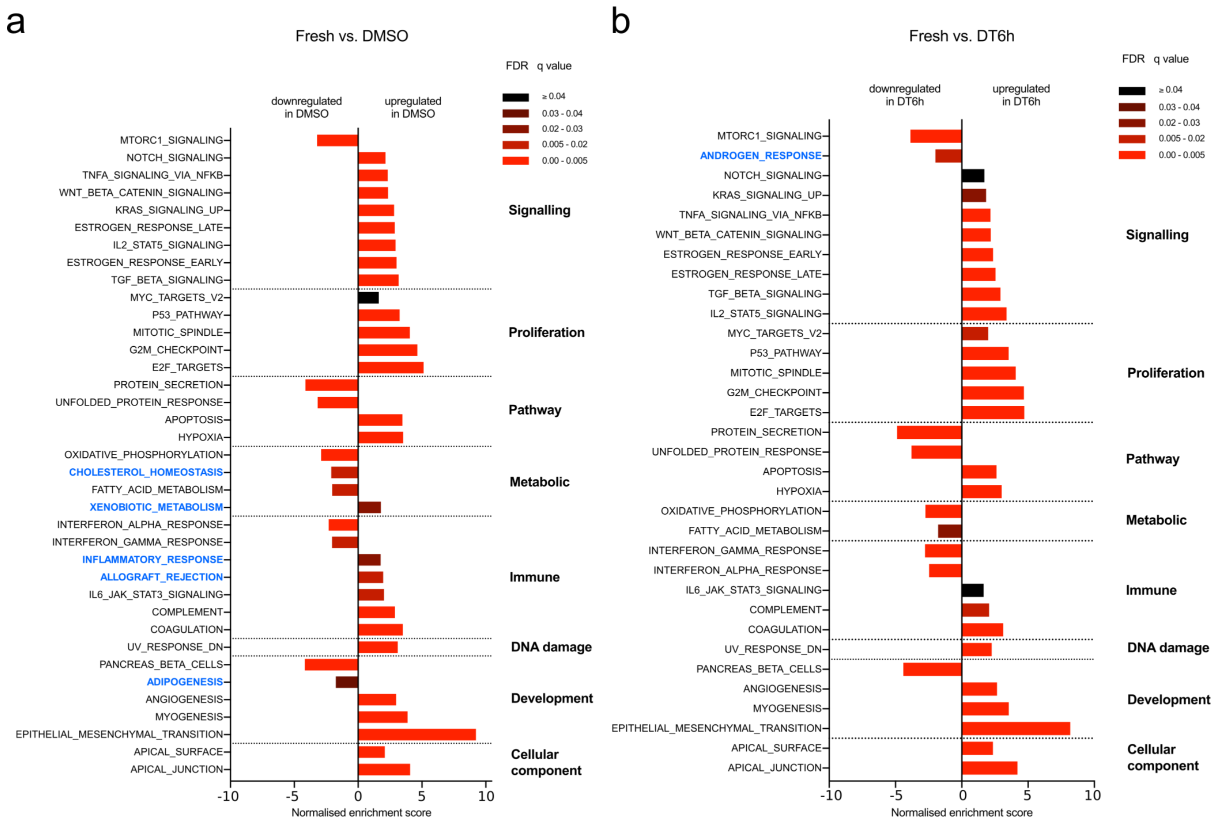

Supplementary Figure 6: Gene Set Enrichment Analysis (GSEA). (a) Preranked Gene Set Enrichment Analysis (GSEA-Preranked) of differences in Hallmark gene sets expression levels (differences of FDR ≤ 0.05 are shown) between fresh and DMSO-cryopreserved islets or **(b)** fresh and DT6h-cryopreserved islets. The gene sets are grouped according to the Process Categories of the Molecular Signatures Database Hallmark Gene Sets (see Methods). Bars are shown in different shades of red based on FDR q-values are shown in different shades of red. Gene sets in (a) which do not appear in (b) and vice versa are highlighted in blue. All plots were created using GraphPad Prism (version 8.4.1, https://www.graphpad.com).

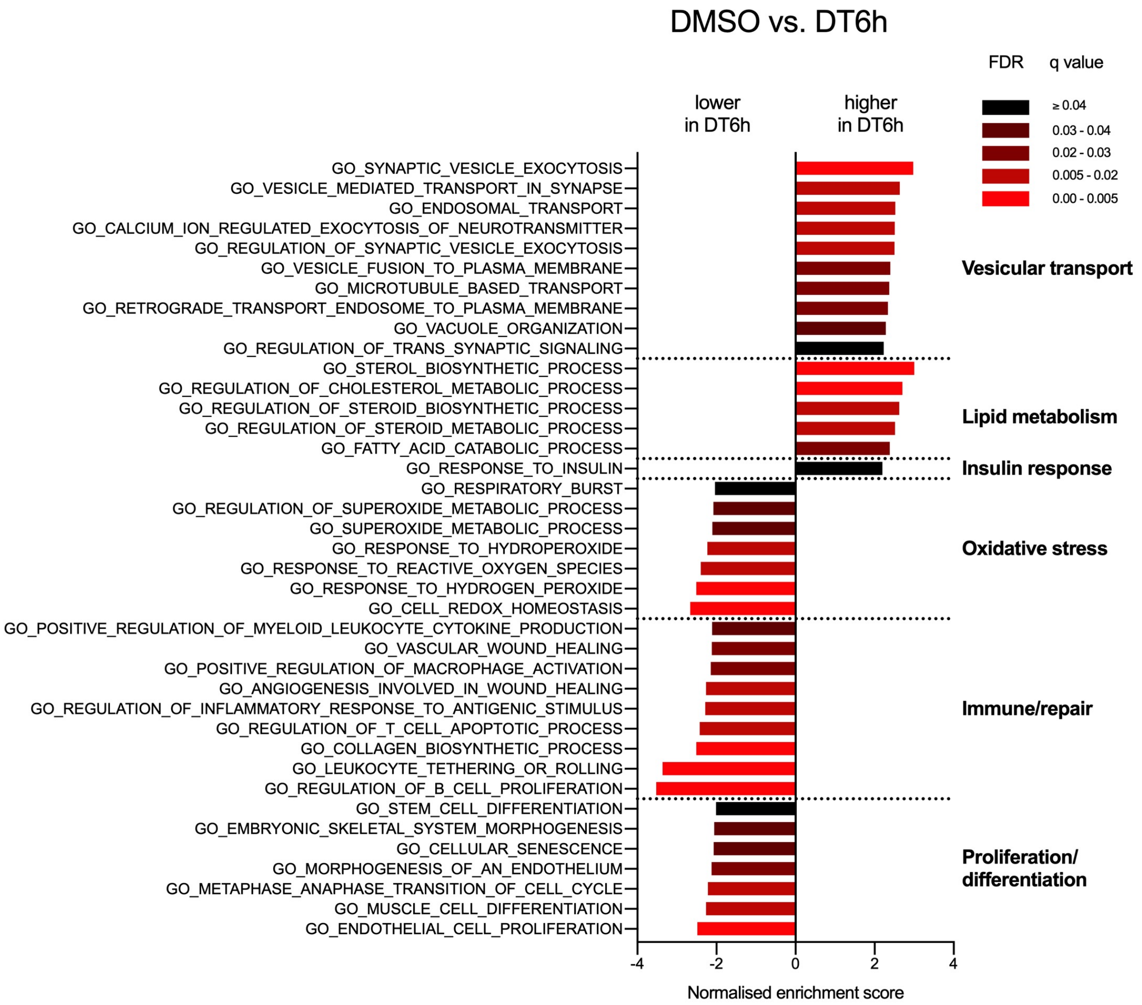

Supplementary Figure 7: Selected pathways from Gene Set Enrichment Analysis (GSEA). Preranked Gene Set Enrichment Analysis (GSEA-Preranked) of relative differences in Gene Ontology: Biological Process gene set expression levels (differences of FDR ≤ 0.05 are shown) between DT6h and DMSO-cryopreserved. Bars are shown in different shades of red based on FDR q-values are shown in different shades of red. All plots were created using GraphPad Prism (version 8.4.1, https://www.graphpad.com).

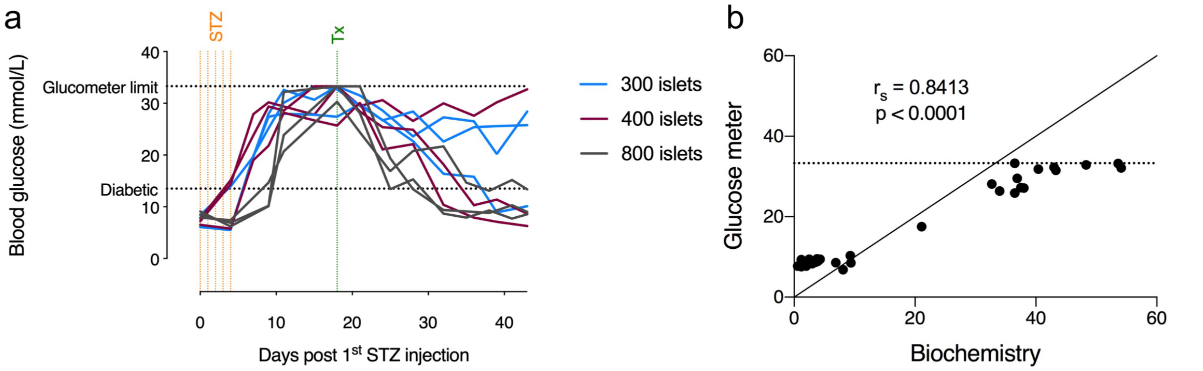

Supplementary Figure 8: Animal experiment optimisation. (a) Non-fasting blood glucose measurements in the study aimed to optimise an optimal dose of mouse islets for transplantation. NSG mice were injected with STZ on 5 consecutive days to induce diabetes and transplanted on day 18 with 300, 400 or 800 fresh mouse pancreatic islets under the kidney capsule. Each line represents one mouse, different colours represent different islet dose. **(b)** Correlation between measurements from the portable glucometer and biochemical assay (n = 38). Line of identity (slope = 1) is displayed on the graph (solid line), as well as the limit of the portable glucose meter (horizontal dashed line). Nonparametric Spearman's correlation coefficient (r_s) was calculated and is displayed together with the p-value. *STZ = streptozotocin, Tx = transplantation*. All plots were created using GraphPad Prism (version 8.4.1, https://www.graphpad.com).

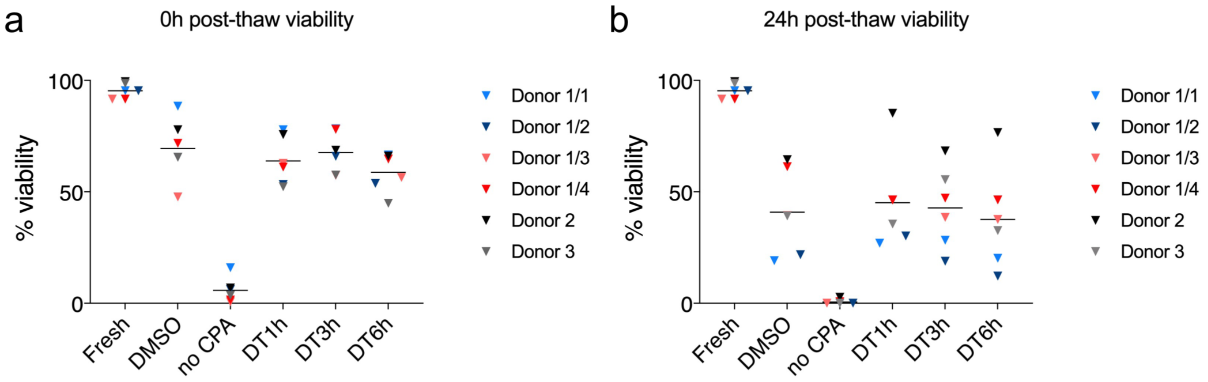

Supplementary Figure 9: Pilot experiments for human islet cryopreservation. Human islets were cryopreserved using the protocol optimised for mouse pancreatic islets, for DMSO + trehalose, three trehalose pre-incubation times (1, 3 and 6 h) are shown. **(a)** Viability of fresh islets and thawed pancreatic islets measured immediately post-thaw. **(b)** Viability of fresh islets and thawed pancreatic islets measured 24 hours post-thaw. Total of 3 human pancreatic islet donors, four sets of experiments from donor no. 1 are shown. Each triangle represents the mean viability of the islets in one experiment. *no CPA = no cryoprotectant*. *DT = DMSO + trehalose*. All plots were created using GraphPad Prism (version 8.4.1, https://www.graphpad.com).
